# On the Microstructure and Properties of Nb-12Ti-18Si-6Ta-2.5W-1Hf (at.%) Silicide-Based Alloys with Ge and Sn Additions

**DOI:** 10.3390/ma13071778

**Published:** 2020-04-10

**Authors:** Jiang Zhao, Claire Utton, Panos Tsakiropoulos

**Affiliations:** Department of Materials Science and Engineering, Sir Robert Hadfield Building, The University of Sheffield, Mappin Street, Sheffield S1 3JD, UK; zhaojiang6325@hotmail.com (J.Z.); c.utton@sheffield.ac.uk (C.U.)

**Keywords:** Nb-silicide-based alloys, high entropy alloys, complex concentrated alloys, microstructures, oxidation, intermetallics, silicides, creep

## Abstract

In this paper two Nb-silicide-based alloys with nominal compositions (at.%) Nb-12Ti-18Si-6Ta-2.5W-1Hf-2Sn-2Ge (JZ1) and Nb-12Ti-18Si-6Ta-2.5W-1Hf-5Sn-5Ge (JZ2) were studied. The alloys were designed using the alloy design methodology NICE to meet specific research objectives. The cast microstructures of both alloys were sensitive to solidification conditions. There was macro-segregation of Si in JZ1 and JZ2. In both alloys the βNb_5_Si_3_ was the primary phase and the Nb_ss_ was stable. The A15-Nb_3_X (X = Ge,Si,Sn) was stable only in JZ2. The Nb_ss_+βNb_5_Si_3_ eutectic in both alloys was not stable as was the Nb_3_Si silicide that formed only in JZ1. At 800 °C both alloys followed linear oxidation kinetics and were vulnerable to pesting. At 1200 °C both alloys exhibited parabolic oxidation kinetics in the early stages and linear kinetics at longer times. The adhesion of the scale that formed on JZ2 at 1200 °C and consisted of Nb and Ti-rich oxides, silica and HfO_2_ was better than that of JZ1. The microstructure of JZ2 was contaminated by oxygen to a depth of about 200 μm. There was no Ge or Sn present in the scale. The substrate below the scale was richer in Ge and Sn where the NbGe_2_, Nb_5_(Si_1-x_Ge_x_)_3_, W-rich Nb_5_(Si_1-x_Ge_x_)_3_, and A15-Nb_3_X compounds (X = Ge,Si,Sn) were formed in JZ2. The better oxidation behavior of JZ2 compared with JZ1 correlated well with the decrease in VEC and increase in δ parameter values, in agreement with NICE. For both alloys the experimental data for Si macrosegregation, vol.% Nb_ss_, chemical composition of Nb_ss_ and Nb_5_Si_3_, and weight gains at 800 and 1200 °C was compared with the calculations (predictions) of NICE. The agreement was very good. The calculated creep rates of both alloys at 1200 °C and 170 MPa were lower than that of the Ni-based superalloy CMSX-4 for the same conditions but higher than 10^−7^ s^−1^.

## 1. Introduction

Ni-based superalloys, which are currently the metallic materials of choice for the hottest parts in state-of-the-art gas turbine engines, operate at temperatures greater than 90% of their melting temperatures assisted by cooling and coatings systems that cause loss of thermal efficiency [1]. These materials have reached their upper temperature limit, which is imposed by the melting point of Ni. New metallic materials with good long-term microstructural stability and load-bearing capabilities at higher turbine entry temperatures are needed to enable future aero-engines to meet performance and emission targets [1,2]. Nb-silicide-based alloys, also known as Nb-silicide in situ composites, are strong contenders owing to their lower densities, significantly higher solidus temperatures, attractive creep and strength properties at high temperatures, and significantly better oxidation resistance compared with conventional Nb alloys. Like the Ni-based superalloys, they are dependent on specific simple, transition (TM) and refractory (RM) metal and metalloid element alloying additions to give a balance of properties [3,4,5,6]. Other candidate metallic materials are Mo-silicide-based alloys. The latter and engineering ceramics or ultra-high temperature ceramics are not considered in this paper. Since 2010, refractory metal high entropy alloys (RHEA) or complex concentrated alloys (RCCA) or multi-principal element alloys (RMPEA) also have been considered as alternative metallic materials for the “beyond the Ni-superalloys” era [7]. The terms CCA or MPEA were proposed and are nowadays used for alloys that do not meet the “accepted” “standard definition” of high entropy alloys (HEA), namely “HEAs are alloys with many principal elements with the concentration of each element being between 35 and 5 at.%” [7,8]. Examples of RCCAs or RMPEAs are the alloys NbTiV_2_Zr [9], Al_0.5_CrNbTi_2_V_0.5_ [10], Al_2_Nb_3_TaTi_3_Zr [8], AlCrMoSi_0.05_TaTi [11] and Al_9.2_Cr_5.7_Hf_0.5_Mo_1.3_Nb_47_Ti_25_V_9.5_W_0.8_ [12]. Some Nb-silicide-based alloys also satisfy the standard definition of HEA [13]. 

The microstructures of Nb-silicide-based alloys consist of alloyed bcc Nb solid solution(s) (Nb_ss_) and intermetallics [13,14] and references within. The latter include tetragonal M_5_Si_3_ and M_3_Si silicides, hexagonal M_5_Si_3_ silicides (M = TM,RM), C14 Laves and A15 phases [13]. The former can be Ti-rich Nb_ss_, Si-free Nb_ss_ and “normal” Nb_ss_ [13] (see next section). Refractory metals confer solid solution strengthening to Nb solid solution [15,16,17,18] and are essential additions for achieving high temperature strength and creep property goals in Nb-silicide-based alloys [4,13,18]. Some Si-free Nb_ss_ in the latter also satisfy the accepted definition of HEA [14]. The strengthening effect of W in Nb is greater than those of Mo or Ta [15,16]. Nb-silicide alloy development research has studied the effects of Mo and/or W on the microstructure and high temperature strength [4,18,19,20]. Only a small number of papers has considered the effects of Ta addition [13] and to our knowledge there is no data for Nb-silicide-based alloys with Ta and W additions. Phases in RHEA/RCCA/RMPEA are one (or sometimes two) bcc (RM)_ss_, and Laves and/or M_5_Si_3_ silicide(s) [8]. In this paper we refer to these materials as RCCAs.

Many elements contribute to improving the oxidation resistance of Nb-silicide-based alloys. These include Al, B, Ce, Cr, Ge, Fe, Hf, Mo, Si, Sn, and Ti [3,5,13,21,22,23] and references within. Pest oxidation at intermediate temperatures (600–900 °C) is suppressed by the synergy of Al, Cr, Si, and Ti with B, Ge, or Sn [3,13]. Spallation of oxide scales formed at high temperatures is a common phenomenon [22,24,25,26]. However, scale spallation was prevented in a Nb-silicide-based alloy when Ge and Sn were added simultaneously with Al, Cr, Hf, Si, and Ti [27]. To date, alloy development has not discovered Nb-silicide-based alloys that meet both creep and oxidation property goals [13,23] and references within. Nb-silicide-based alloys, like the Ni-based superalloys, will require environmental coating system(s) yet should have inherent oxidation resistance to survive in case of coating failure [23]. Elements in RCCAs are the group IV, V, and VI TMs and RMs and sometimes there are additions of Al and/or Si (and Co or Ni). The same elements are used as additions in Nb-silicide-based alloys [13] with the exception of Ta, V, or Zr that have been used less frequently and Co and Ni that have not been used at all. 

Toughness at room temperature, oxidation in the pest regime (600–900 °C) and at high temperatures (≥1000 °C), and creep at T ≥ 1050 °C and stress (σ) in the range 50 to 300 MPa have been evaluated for Nb-silicide-based alloys [2,3,4,5,13,18,19,20,21,22,23,24,25,26,27,28,29] and references within. Properties of the key phases in the latter alloys have also been measured [13] and references within and the effects of alloying on properties of Nb_5_Si_3_ have been studied [13] and references within. In contrast, creep properties and pest oxidation of RCCAs have not been studied [8]. Yield strength of RCCAs has been measured in compression tests at room temperature and 1000 °C, very few RCCAs have been tested in tension tests at room temperature, few RCCAs were tested in compression at 1200 °C and only two RCCAs have been tested at 1400 and 1600 °C [8]. The oxidation of RCCAs has been reported mainly at 1000 °C with few studies covering higher temperatures (1100, 1300 °C) and even fewer studies at lower temperatures [8]. The lack of oxidation studies of RCCAs in the pest regime is noticeable. 

Contamination by interstitial elements is a serious issue in refractory metal alloys [15,16,17]. The contamination of phases by oxygen in Nb-silicide-based alloys in the bulk and below the scale has been studied systematically in our group, for example see [26,27]. There is a noticeable lack of studies for the contamination of phases by oxygen in RCCAs. The significance of interstitial contamination for the mechanical properties of RCCAs has been highlighted in [8]. 

Refractory metal additions are essential for the creep and high temperature strength of Nb-silicide-based alloys [4,13,18] and references within. Creep resistant Nb-silicide-based alloys should not suffer from pest oxidation and scale spallation. Could these aims be achieved in alloys with simultaneous additions of RMs, Ge, and Sn? The motivation of the research presented in this paper was to provide answers to this question. In this paper we study how the simultaneous addition of Ta, W, Hf, Ge, and Sn affects the microstructure and oxidation of two Nb-silicide-based alloys without Al and Cr. The structure of the paper is as follows. First, the design and selection of the alloys using the alloy design methodology NICE [13] is discussed. The experimental details are followed by the results for the as-cast and heat-treated microstructures and the isothermal oxidation of the alloys at 800 and 1200 °C. The discussion considers first the Si macrosegregation in the alloys, then their solidification and heat-treated microstructures, and this is followed by discussion of their oxidation. Predicted microstructures and properties using NICE [13] are compared with the experimental data.

## 2. Alloy Design and Selection

Nb-silicide-based alloys can be separated in three groups, depending on alloying additions and “targeted property” [6]. Alloys with transition/refractory metal additions meet (are close to) the creep property goal, which is “the creep strength should be greater than 170 MPa at a creep rate of 2 × 10^−8^ s^−1^ at 1200 °C” (the creep goal assumes alloy density of 7 g/cm^3^) [13,28], and occupy a particular area in the Δχ or VEC versus δ maps [6]. In the latter, the “basis” alloy YG8 has nominal composition Nb-18Si-5Hf-5Mo-3W [30]. For this alloy the alloy design methodology NICE [13] gives creep rate έ = 4.8 10^−8^ s^−1^ at T = 1200 °C and σ = 170 MPa and experiments gave έ = 10^−7^ s^−1^ at T = 1200 °C and σ = 200 MPa. 

At 1200 °C and 200 MPa, polycrystalline Nb-silicide-based alloys with nominal compositions Nb-18Si-xRM-5Hf and Nb-zTi-18Si-xRM-yHf (RM=Mo,Ta,W, x = 2,3,5, y = 1,5, z = 8,11) [30,31] (i) lie in the area B in the Figure 1 in [6], and (ii) have experimental creep rates in the range 1.1 × 10^−6^ to 1.1 × 10^−8^ s^−1^, compared with creep rates in the range 10^−6^ to 4.4 × 10^−8^ s^−1^ predicted by NICE [13] for T = 1200 °C and σ = 170 MPa. At 1200 °C, the experimental creep rates of the single crystal CMSX-4 Ni-based superalloy are 2 × 10^−6^ s^−1^ and 6.3 × 10^−5^ s^−1^, respectively at 100 and 150 MPa. To our knowledge, CMSX-4 is not used under the above conditions.

In synergy with Al and/or Cr, Sn or Ge improve the oxidation resistance of Nb-silicide-based alloys in the pest oxidation regime when added individually [3,13,24,25,26] and in the pest regime and at higher temperatures when added simultaneously [3,27]. There is no data about (i) the microstructure and (ii) the oxidation of Nb-silicide-based alloys in which Ge and Sn are added simultaneously with TMs and RMs but without Al and Cr additions. Two objectives of the research presented in this paper are to elucidate (i) and (ii).

In Nb-silicide-based alloys three types of bcc Nb solid solution can form, namely “normal” Nb_ss_, Ti-rich Nb_ss_, and Nb_ss_ with no Si (i.e., Si-free Nb_ss_, see above) [14]. The latter is observed in alloys with Mo, Ta, or W additions [14,30,31] but is stable only when Mo or Mo and W are present in the alloy [30,31]. In the Nb solid solution the partitioning of Ti is opposite to that of Mo or W, meaning Ti-rich Nb_ss_ tends to be poor in Mo or W and vice versa [31]. There is no data (iii) about the partitioning of Ti, Ta, and Mo or W in the Nb solid solution and (iv) about the type(s) of Nb solid solution(s) that is(are) stable in Nb-silicide-based alloys with simultaneous addition of RMs, Sn, and Ge. Two additional objectives of this paper are to study (iii) and (iv). 

We used the alloy design methodology NICE [13] to select two Nb-Ti-Si-Ta-W-Hf-Ge-Sn silicide-based alloys. The starting point of alloy design was the target for creep rate έ = 10^−7^ s^−1^ at T = 1200 °C and σ = 170 MPa [13]. Alloy compositions were selected as described in [13] with specific constraints about alloying additions. The latter were as follows: the alloys should 

(a) Lie in maps of the parameters δ, Δχ, and VEC in the area of Nb-silicide-based alloys with TM and/or RM additions that meet or are close to the creep property goal (see above);

(b) Be free of Al and Cr (because of objectives (i) and (ii));

(c) Have lower density than state-of-the-art Ni-based superalloys (ρ ≈ 9 g/cm^3^ for 3rd generation, ρ ≈ 8.64 to 8.95 g/cm^3^ for 2nd generation [1]) and lower than the density of single-phase bcc solid solution Al and Cr-free RCCAs with Ta additions (ρ ≈ 8.96 to 13.6 g/cm^3^ [8]); 

(d) Have Ta and W additions with Ta/W = 2.4, which is higher than the ratio in RCCAs studied to date (Ta/W ≤ 1 [8]) and to keep low the DBTT (owing to the strong negative effect of W compared with Ta [16]);

(e) Ti and Hf additions with Ti/Hf = 12, which is higher than the ratio in RCCAs studied to date (Ti/Hf ≤ 3 [8]) and to keep low the DBTT [16]; 

(f) Sn/Ge = 1 and (g) Sn concentrations of 2 or 5 at.%. 

As regards the above constraints, the (a), (d), and (e) were related to the creep target and data about creep in [4,13] and references within, the (b), (d), (e), and (g) were linked with (c) and the aim to have specific strengths higher than Ni-based superalloys, and the (b), (f), and (g) were linked with oxidation resistance. The choice of Sn concentrations, and thus constraints (f) and (g), was also “guided” by literature about the effect of Sn or Ge on the oxidation behavior of Nb-silicide-based alloys [3,13,24,25,26,27]. 

The nominal compositions (at.%) of the selected alloys were Nb-12Ti-18Si-6Ta-2.5W-1Hf-2Sn-2Ge (alloy JZ1) and Nb-12Ti-18Si-6Ta-2.5W-1Hf-5Sn-5Ge (alloy JZ2). In addition to the objectives (i) to (iv) that were given above, other objectives of the research presented in this paper were to compare (v) the predicted by NICE [13] creep rates for the actual compositions of the alloys JZ1 and JZ2 with the target one (see above), and (vi) the predicted by NICE [13] composition and vol.% of the Nb_ss_ in each alloy with the actual compositions and volume fractions of the solid solution. Related with the objective (ii), we also wish to compare (vii) with that predicted by NICE [13] and measure the weight gains after isothermal oxidation at 800 and 1200 °C for the actual compositions of the alloys JZ1 and JZ2. Objectives specific to solidification processing were (ix) to find out whether the alloys JZ1 and JZ2 could be prepared using arc melting, owing to them having alloying additions with a very wide range of melting temperatures (T_m_^Sn^ = 232 °C, T_m_^Ge^ = 937 °C, T_m_^Si^ = 1412 °C, T_m_^Ti^ = 1667 °C, T_m_^Hf^ = 2227 °C, T_m_^Nb^ = 2467 °C, T_m_^Ta^ = 2980 °C and T_m_^W^ = 3400 °C) and (x) to compare that predicted by NICE [13] macrosegregation of Si (MACSi) with the measured MACSi in each alloy.

## 3. Experimental

The two alloys were produced from high-purity elements (better than 99.99 wt.%) using arc melting under an argon atmosphere in a water-cooled copper crucible with a non-consumable tungsten electrode. Each alloy was melted five times to ensure as much as possible chemical homogeneity and cooled in the water-cooled copper crucible. Control of alloy composition proved very difficult owing to the loss by evaporation of elements with low melting points. Elemental losses were compensated by increasing the weight of each element that was lost. After many attempts, the “best” two alloys with composition as close as possible to the nominal ones were selected for further study. These are the alloys reported in this paper. The alloys were richer and poorer respectively in Si and Sn compared with the nominal compositions (see next section).

The alloy buttons were sectioned to produce specimens containing the top, bulk, and bottom areas of the button (bottom refers to the side facing the water-cooled copper crucible during melting) in order to fully characterize their as-cast microstructures and in particular to find out if there were different microstructures in the parts of the buttons that solidified under high cooling rates. The specimens were mounted in Bakelite, ground using 120, 400, 800, and 1200 grit papers, and polished to 1 μm surface finish using 6 μm, 3 μm, and 1 μm diamond pastes.

There were no peaks associated with the melting in each alloy up to 1600 °C, the highest temperature used in differential scanning calorimetry (DSC) experiments (data not shown). Specimens for heat treatment were cut from the bulk of the buttons, wrapped in Ta foil, placed in an alumina crucible, and heat treated at 1500 °C for 100 h in a tube furnace under a flow of argon. These conditions are the same with the highest temperature and longest time used in research on Nb-silicide-based alloys [30,31]. Titanium sponge was used at the entrance of the argon flow as the oxygen getter. The specimens were furnace cooled.

A Siemens D5000 X-ray diffractometer (HiltonBrooks Ltd., Crew, UK) with monochromatic CuKα radiation (λ = 1.540562 Å) was used to identify the phases in as-cast and heat-treated specimens. Phase identification was done by matching the characteristic peaks in the XRD diffractogram with PDF (powder diffraction file) data using the ICDD PDF-4+ (International Centre Diffraction Data, Newton Square, PA, USA) and Sieve+ software. 

The microstructures of the alloys were observed using scanning electron microscopy (SEM) with backscattered electron (BSE) imaging and energy dispersive X-ray spectrometry (EDS). The latter was used to determine the chemical composition of each alloy and the phases in its microstructure. Inspect F SEM, JEOL 6400 SEM (JEOL Ltd., Tokyo, Japan) and Philips XL 30S FEG SEM instruments (ThermoFisher Scientific, Hillsboro, OR, USA) were used to study the microstructures. Chemical compositions of large areas from the top, bulk, and bottom of the buttons and of constituent phases were analyzed using JEOL 6400 SEM and Philips XL 30S FEG SEM instruments under a voltage of 20 kV. For the EDS, specimens of high purity Nb, Ti, Si, Hf, Ta, W, Ge, Sn, and Al_2_O_3_ that were polished to 1 μm finish were used as standards. Calibration of the EDS detector (Oxford Instruments, High Wycombe, UK) was done using a specimen of pure Co and was repeated every hour during analysis. A minimum of five EDS analyses of large areas and phases were performed. Only phases with size larger than 5 μm were analyzed. The chemical analysis data is given with the average, minimum, and maximum values and standard deviation. Area fractions of Nb_ss_ were calculated using the software Image-Pro with images of microstructures taken in the SEM in BSE imaging mode.

Thermogravimetric (TG) analysis was used to study the isothermal oxidation of the alloys at 800 °C and 1200 °C for 100 h. For the oxidation experiments, cubic samples of 3 × 3 × 3 mm^3^ were cut from the as-cast alloys and their sides were ground to 1200 grit. The dimensions of each specimen were measured using a micrometer and the surface area was calculated. Each specimen was placed in a small alumina crucible and its oxidation was studied in a NETZSCH STA 449 F3 thermal analyzer (NETZSCH GmbH, Selb, Germany). The rate of 3 °C per minute was applied in both heating and cooling. The oxidized samples were cold mounted and polished. Inspect F SEM and Philips XL 30S FEG SEM instruments were used to study the microstructures from the oxide scale to the bulk. The density of the alloys was measured using an AccuPyc II 1340 gas pycnometer. 

## 4. Results

### 4.1. Alloy JZ1

**Figure 1:**
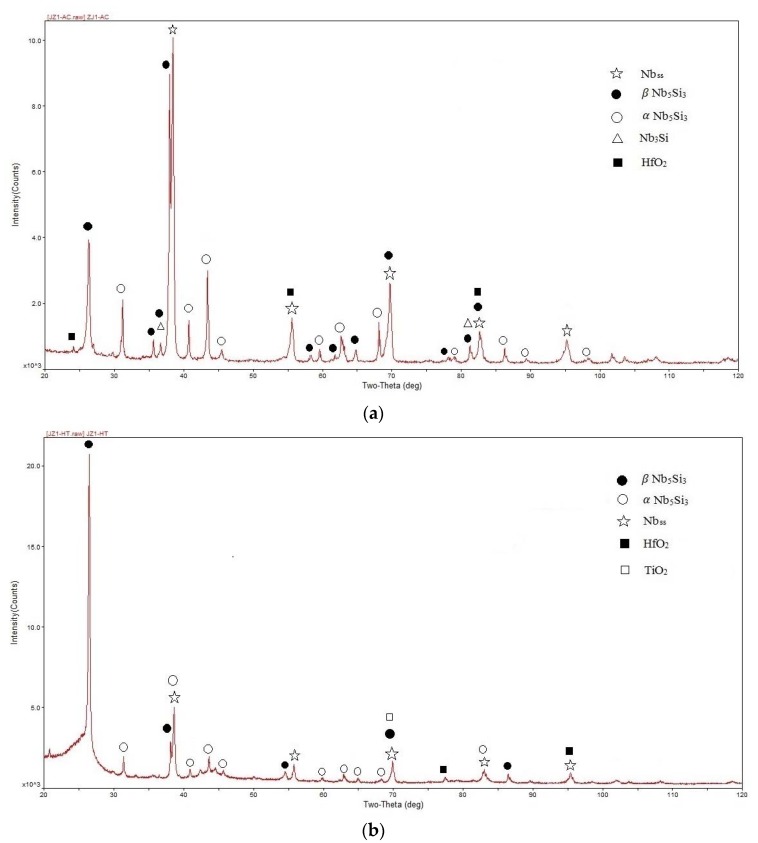
X-ray diffractograms of the (**a**) as-cast and (**b**) heat-treated alloy JZ1.

The actual composition of the as-cast alloy (JZ1-AC) was 55Nb-11.6Ti-22.5Si-5.3Ta-1.7W-0.9Hf-1.2Sn-1.8Ge. This was the average of all the large area analyses taken from the top, bulk, and bottom of the button. There was macrosegregation of Si, the concentration of which was in the range 19 at.% to 24.6 at.%. The XRD and EDS data (Figure 1 and Table 1) confirmed the Nb_5_Si_3_, Nb_3_Si, Nb_ss_, and HfO_2_ phases in the microstructure of JZ1-AC. The Nb_5_Si_3_ existed in both the βNb_5_Si_3_ and αNb_5_Si_3_ structures (Figure 1). The Nb_3_Si silicide was observed only in the bottom of the button.

**Figure 2:**
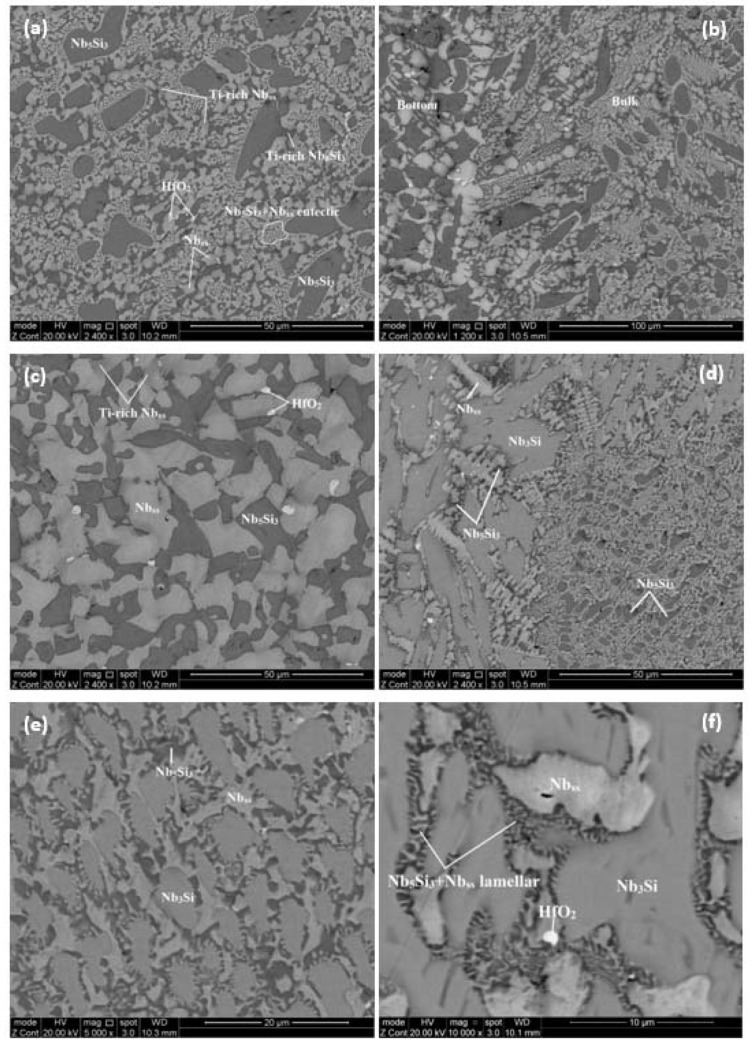
Backscattered electron (BSE) images of the microstructure of JZ1-AC in the (**a**) top and (**c**) bottom (**b**) showing the transition from the bulk to the bottom. (**d**) to (**f**) show the details of the microstructure in the bottom of the button of JZ1-AC where the Nb_3_Si silicide was observed.

The typical microstructures are shown in Figure 2. They did not differ in the top and bulk of the button, and consisted of irregular large Nb_5_Si_3_ grains surrounded by fine Nb_5_Si_3_ + Nb_ss_ eutectic and Nb_ss_ and a small vol.% of HfO_2_ (Figure 2a). The vol.% of the Nb_ss_ in the top was lower than in the bulk (Table 2). Tungsten and Sn partitioned to the Nb_ss_, Ge to the Nb_5_Si_3_ and Ta to both phases. The areas exhibiting darker contrast in the Nb_5_Si_3_ and Nb_ss_ were rich in Ti and had lower concentrations of Ta and W and higher contents of Sn, Ge (only for Nb_5_Si_3_), and Hf, compared with the “normal” Nb_5_Si_3_ and Nb_ss_. In the Nb_5_Si_3_ and Ti-rich Nb_5_Si_3_, the sums of the Si + Sn + Ge concentrations were 39.1 at.% and 37.9 at.%, respectively. The average composition of the Nb_5_Si_3_ + Nb_ss_ eutectic was 58.8Nb-10.8Ti-18.2Si-6.2Ta-2.8W-0.7Hf-1.3Sn-1.2Ge with Si + Sn + Ge = 20.7 at.%.

The microstructure in the bottom of the button contained only of Nb_5_Si_3_ and Nb_ss_ without the Nb_5_Si_3_ + Nb_ss_ eutectic (Figure 2c). The vol.% of Nb_ss_ in the bottom was the same as in the bulk (Table 2). There was a small region in the bottom of the button where the Nb_3_Si was present, surrounded by Nb_5_Si_3_ + Nb_ss_ lamellar microstructure and Nb_ss_ (Figure 2d–f). The average composition of this area was 56Nb-11.8Ti-21.2Si-5.6Ta-1.8W-1Hf-1.3Sn-1.3Ge with Sn/Ge = 1, slightly poorer in Si and Ge and richer in Ta and Sn, compared with the rest of the button. Unlike the Nb_5_Si_3_, in the Nb_3_Si the concentrations of Ta and W were relatively high, respectively at 6.5 at.% and 1.5 at.%, and the Sn solubility was very low. 

**Figure 3:**
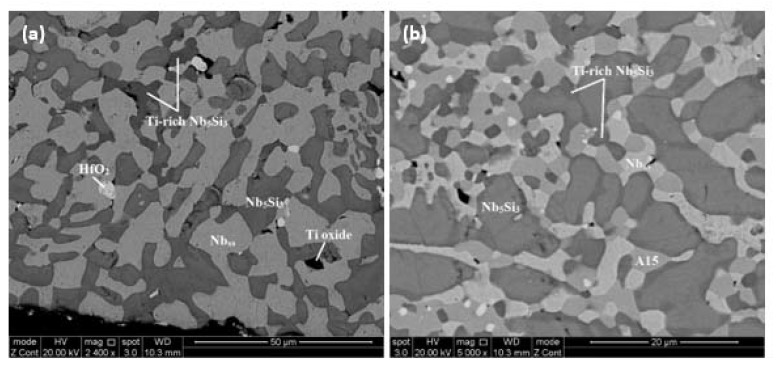
BSE images of the microstructures of the heat-treated alloys (**a**) JZ1 and (**b**) JZ2.

The average composition of the alloy after the heat treatment (JZ1-HT) was 55Nb-12Ti-22.4Si-5.2Ta-1.6W-0.8Hf-1.2Sn-1.8Ge. There was still Si inhomogeneity in the microstructure. The microstructure consisted of Nb_5_Si_3_, Nb_ss_, HfO_2_, and Ti oxide (Figure 3a, Table 1). According to the XRD data (Figure 1b), the Nb_5_Si_3_ existed in both the αNb_5_Si_3_ and βNb_5_Si_3_ forms with more peaks corresponding to the former. There were no peaks corresponding to the Nb_3_Si. EDS showed there was no Ti-rich Nb_ss_, whereas Ti-rich Nb_5_Si_3_ was still present. The vol.% of the Nb_ss_ had not changed after the heat treatment (Table 2).

Compared with the JZ1-AC, there were slight changes in the concentration of Si, Ta, W, Sn, and Ge in the Nb_ss_ and the concentration of Hf in the Nb_ss_ was reduced to zero owing to the consumption of Hf to form HfO_2_. The Ta + W content in the Nb_ss_ was reduced to 12.0 at.%. The composition of the Nb_5_Si_3_ was essentially unchanged and there was no W in the Ti-rich Nb_5_Si_3_. Titanium oxide had formed just below the surface of the specimen because of the contamination of the latter by oxygen during the heat treatment.

### 4.2. Alloy JZ2

**Figure 4:**
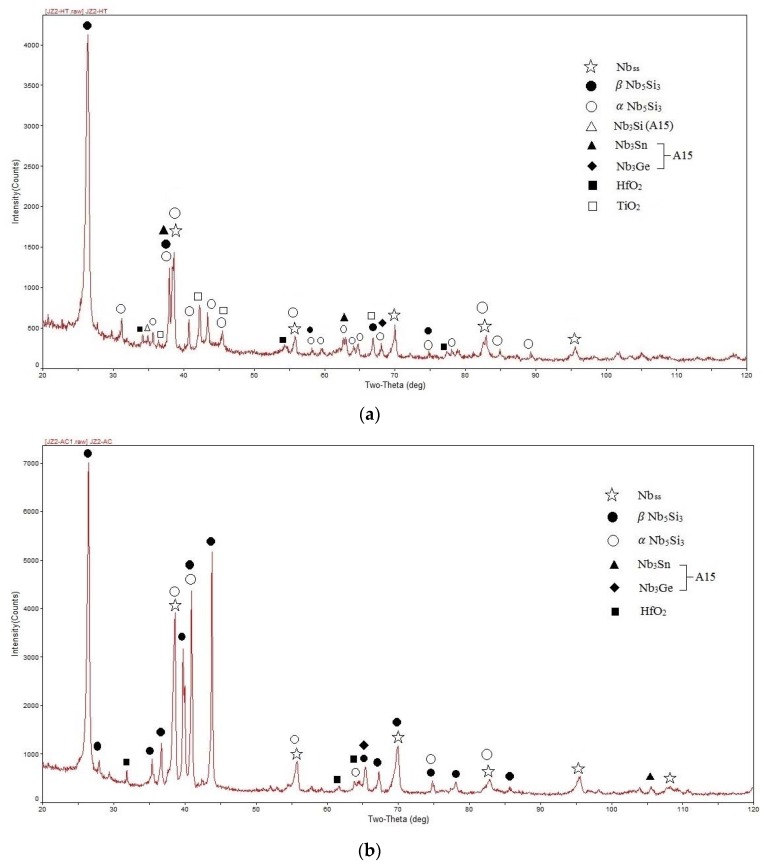
X-ray diffractograms of the (**a**) as-cast and (**b**) heat-treated alloy JZ2.

The actual composition of the as-cast alloy (JZ2-AC) was 50.5Nb-11.8Ti-21.5Si-5.2Ta-2.0W-1Hf-2.8Sn-5.2Ge. This was the average of all the analyses taken from the top, bulk, and bottom of the button. There was macrosegregation of Si with the Si content in the range of 18.8 to 23.7 at.%. The XRD and EDS data (Figure 4 and Table 3) confirmed the Nb_5_Si_3_, Nb_ss_, A15 compounds and HfO_2_. Both the βNb_5_Si_3_ and αNb_5_Si_3_ were present in the diffractogram with more peaks of the former. The A15 compound was observed only in the bottom of the button.

**Figure 5:**
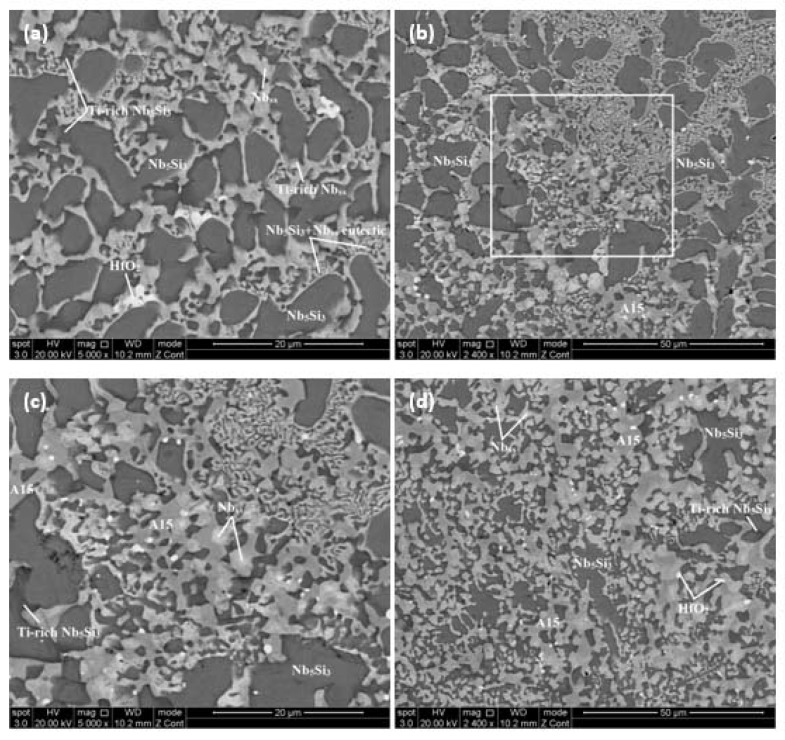
BSE images of the microstructure of JZ2-AC in (**a**) bulk, (**b**) and (**c**) the transition from the bottom to the bulk and (**d**) bottom of the button. (**c**) Corresponds to the area shown by square in (**b**).

The microstructure is shown in Figure 5. In the top and bulk of the button the primary Nb_5_Si_3_ was surrounded by Nb_5_Si_3_ + Nb_ss_ eutectic, Nb_ss_ and HfO_2_. The average composition of the eutectic was 53.2Nb-13.5Ti-15.6Si-5.9Ta-3W-3.4Sn-4.3Ge-1.1Hf with Si + Ge + Sn = 23.3 at.%. Compared with JZ1-AC, the eutectic was richer in Ti and poorer in Si. In the bottom there were A15, finer Nb_5_Si_3_ and Nb_ss_ and no eutectic and Nb_3_Si. The top right hand corner of Figure 5c shows the change from the bottom microstructure to that in the bulk. The partitioning of elements between the Nb_ss_ and the Nb_5_Si_3_ was the same as in JZ1-AC. Titanium-rich Nb_ss_ and Nb_5_Si_3_ grains were observed in the top and bulk but no Ti-rich Nb_ss_ was formed in the bottom of the button. The enrichment of Ti in the solid solution and the silicide “pushed out” the Ta and W and drew in the Sn in the Nb_ss_ and the Sn, Ge, and Hf in the Nb_5_Si_3_. In the Nb_ss_ and Ti-rich Nb_ss_, the Ta + W content was 15.8 at.% and 12.0 at.%, respectively, the latter significantly higher compared with the alloy JZ1-AC (7.3 at.%). The Si + Sn + Ge concentration in the “normal” and Ti-rich Nb_5_Si_3_ were 39.5 at.% and 38.7 at.%. The vol.% of the Nb_ss_ in the bottom was lower than those in the top and bulk (Table 2), owing to the presence of A15. The latter exhibited darker contrast than the Nb_ss_ in BSE images. The Si + Sn + Ge and Ta + W contents in the A15 were 19.2 at.% and 6.9 at.%, respectively. 

The average composition of the alloy after the heat treatment (JZ2-HT) was 51Nb-12Ti-21.1Si-5.4Ta-2.1W-0.8Hf-2.7Sn-4.9Ge. Chemical inhomogeneity of Si was still present. The typical microstructure is shown in Figure 3b. The XRD and EDS data (Figure 4b and Table 3) confirmed the presence of A15, Nb_ss_, Nb_5_Si_3_, and TiO_2_. The eutectic microstructure was not stable and the Nb_5_Si_3_ existed with both the β and α structures with fewer peaks corresponding to the former (Figure 4b).

In the Nb_ss_ the Si solubility was high (3.5 at.%) and the Sn concentration had decreased to 2.2 at.%. The concentrations of Ta and W in the Nb_ss_ had increased and the Ta + W content was 17.5 at.%, higher than that in JZ1-HT (12.0 at.%). Ti-rich Nb_5_Si_3_ was still observed and there were Ti-rich sub-grain boundaries in the Nb_5_Si_3_. The latter also were observed in the αNb_5_Si_3_ in heat-treated small buttons of the alloy CM1 (=Nb-8.3Ti-21.1Si-5.4Mo-4W-0.7Hf) in [31]. The chemical compositions of the Nb_5_Si_3_ and the A15 compounds did not change significantly after the heat treatment. Hafnium in the Nb_ss_ and the A15, and W in the Ti-rich Nb_5_Si_3_ were undetectable. Titanium oxide formed below the surface after the heat treatment.

### 4.3. Oxidation

#### 4.3.1. Oxidation at 800 °C 

**Figure 6:**
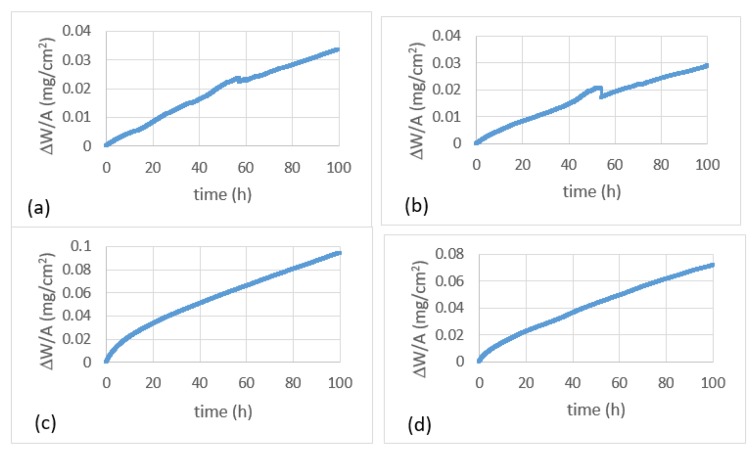
Thermogravimetric (TG) data for the alloys JZ1 (**a**,**c**) and JZ2 (**b**,**d**) for 800 °C (**a**,**b**) and 1200 °C (**c**,**d**).

**Figure 7:**
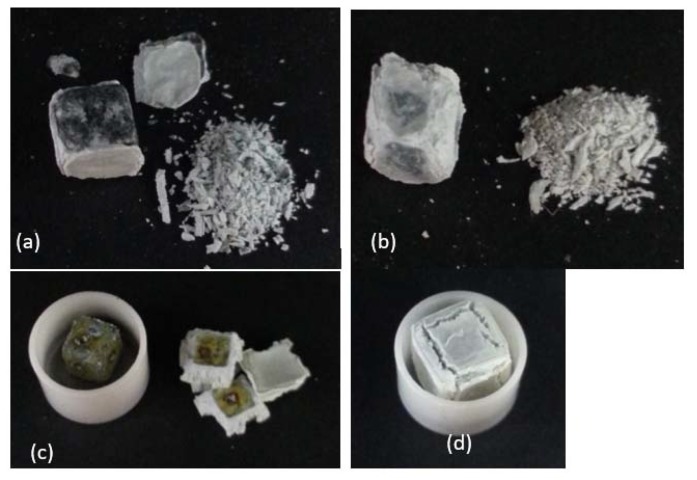
The specimens of the alloys JZ1 (**a**,**c**) and JZ2 (**b**,**d**) after oxidation at 800 °C (**a**,**b**) and 1200 °C (**c**,**d**). The size of each oxidation specimen was 3 × 3 × 3 mm^3^.

The TG data of the alloys are shown in Figure 6a,b and the specimens after the oxidation experiments are shown in Figure 7a,b. Both alloys were susceptible to pest oxidation. The weight gains of the alloys JZ1 and JZ2 after 100 h were 33.6 mg/cm^2^ and 28.9 mg/cm^2^, respectively (Table 4). The weight gains of both alloys increased gradually up to about 40 h. At about 56 and 54 h from the start of the experiment the alloys JZ1 and JZ2, respectively, showed an abrupt drop in weight gain, after which the weight increased linearly at a similar rate as that from the start of the experiment to about 40 h. The oxidation of both alloys followed linear kinetics with similar oxidation rates (Table 4). Typically, a sudden weight loss may be due to the scale spalling and falling from the crucible/balance so that a large weight loss is detected.

#### 4.3.2. Oxidation at 1200 °C

The TG data of the alloys is shown in Figure 6c,d and the specimens are shown in Figure 7c,d. The oxidation of both alloys exhibited parabolic kinetics in the initial stage of oxidation, followed by linear kinetics (Table 4). The weight gain of the alloy JZ1 was 91.3 mg/cm^2^ and there was spallation of its oxide scale. The weight gain of the alloy JZ2 was lower (71.4 mg/cm^2^) and the adherence of its scale was better. Because of the latter, only the cross section of the alloy JZ2 was studied.

**Figure 8:**
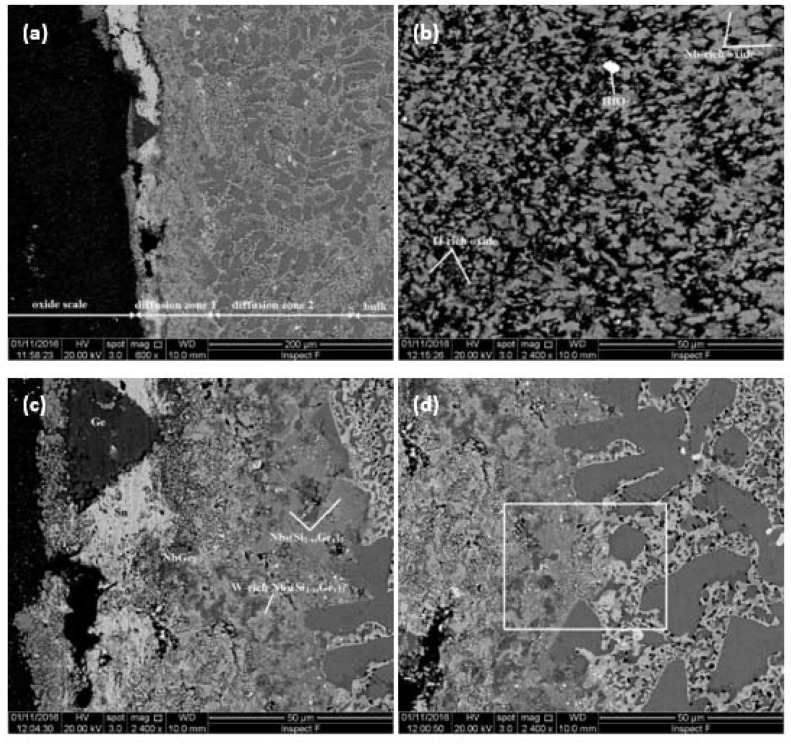
BSE images of the microstructure (**a**) of a cross section of the oxidized alloy JZ2, (**b**) of the oxide scale, (**c**,**d**) of the diffusion zones 1 and 2, respectively. For the region indicated by a rectangle in (**d**), see later on the Figure 10 and text.

A cross section of the alloy JZ2 is shown in Figure 8a. There were three regions, namely the oxide scale, diffusion zone, and bulk. Pores and cracks were observed in the scale. The diffusion zone consisted of two different microstructures, which are marked as diffusion zone 1 and diffusion zone 2 in Figure 8a.

Table 5 gives the compositions of three oxides that were formed in the scale, namely Nb-rich oxide (grey contrast phase), Ti-rich oxide (darker grey contrast phase), and HfO_2_ (white contrast phase). Silica was also present in the scale. It appeared as a black phase and had the same contrast with pores and cracks. No Ge and Sn were detected in the scale. The microstructure in the bulk was similar to that of the cast alloy and included the A15 compound. The Ti-rich Nb_5_Si_3_ silicide and A15 compound were leaner in Si, the former was richer in Ti and the Nb_ss_ was leaner in Sn, compared with JZ2-AC and JZ2-HT. 

The Nb_ss_, Nb_5_Si_3_, A15, HfO_2_, and Ti oxides were present in the diffusion zone 2. The TiO_2_ exhibited black contrast and was formed adjacent to the Nb_5_Si_3_ and inside the Nb_ss_ and A15, where the Ti concentrations were reduced by 4.8 and 2.4 at.%, respectively, compared with the bulk, owing to the consumption of Ti to form the oxide. This indicated the contamination of the microstructure by oxygen to the depth of diffusion zone 2 below the scale (about 200 μm). The phases in the diffusion zone 1 were the Nb_5_(Si_1-x_,Ge_x_)_3_, NbGe_2_, and HfO_2_. There were W-rich areas in the former compound. The tI32-W_5_Si_3_-type Nb_5_Ge_3_ and the NbGe_2_ are stable compounds in the Nb-Ge binary system. The former is isomorphous with βNb_5_Si_3_. Thus, the Nb_5_(Si_1-x_,Ge_x_)_3_ and the W-rich areas of the compound that formed in the diffusion zone 1, most likely were silicides of the W_5_Si_3_-type. The formation of Nb_5_(Si_1-x_,Ge_x_)_3_ in the diffusion zone is in agreement with [27].

**Figure 9:**
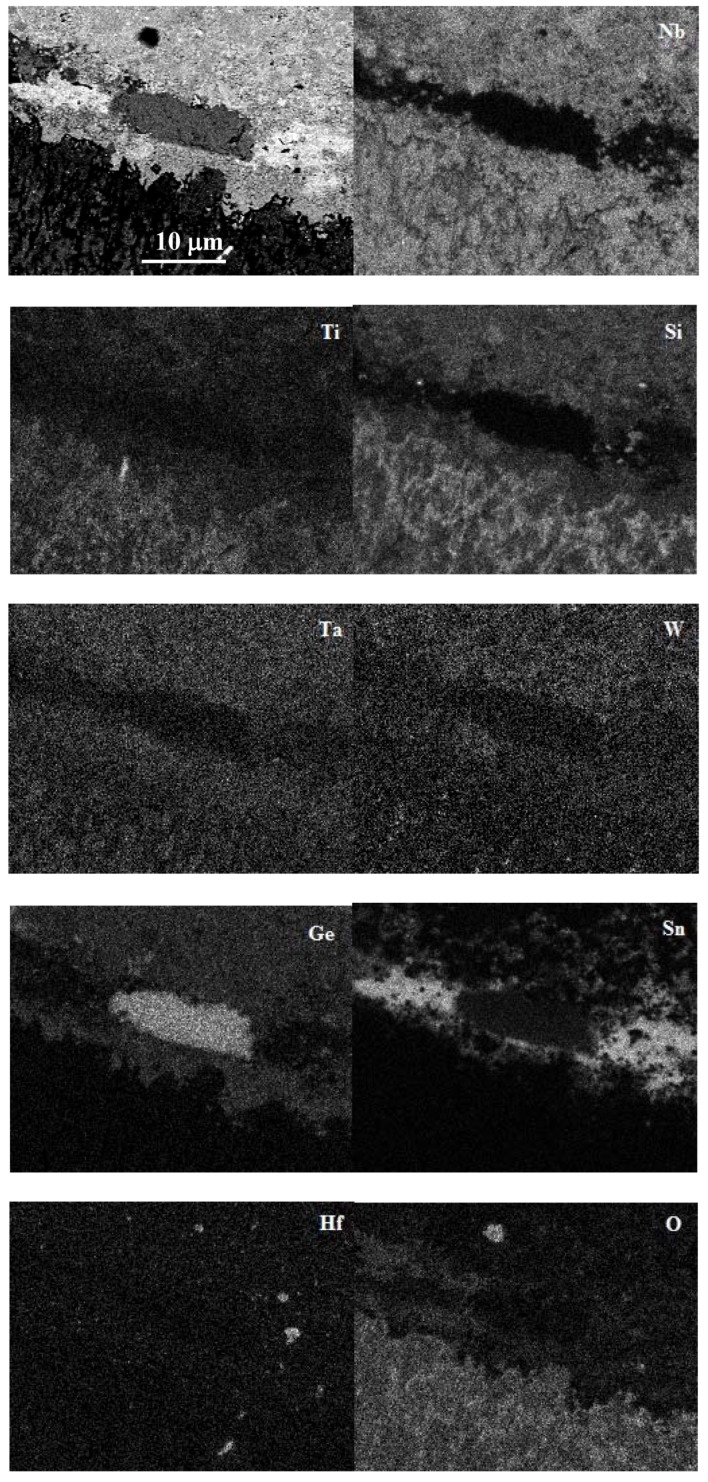
BSE image and X-ray maps of a region in diffusion zone 1.

A continuous layer exhibiting bright contrast that separates the diffusion zone 1 in two parts should be noticed in Figure 8a. The chemical analysis data and the X-ray maps in Figure 9 showed that the layer was rich in Sn and lean in other elements and oxygen. The layer had some regions of dark contrast, which were rich in Ge (Figure 9). In the regions near the interface between the diffusion zones 1 and 2, a high volume fraction of particles of white contrast were dispersed in the Nb_ss_, A15, and Nb_5_(Si_1-x_,Ge_x_)_3_ (Figure 8d). These particles were too small for the chemical analysis but X-ray maps suggested that some were rich in W (Figure 9), most likely W-rich solid solution. The maps in Figure 9 also confirmed the enrichment in Ge and Sn toward the surface of the oxidized specimen.

## 5. Discussion

### 5.1. Densities

The densities of both alloys (Table 2) met the constraint (c) (see Section 2), were lower than the upper density value of TM HEA (9 g/cm^3^ [8]) and multiphase RCCAs with/out Al, Cr, or Si (8.6 g/cm^3^ [8]) and were also lower than (a) the densities of the alloys YG4 (Nb-18Si-5Hf-3Ta-2Mo), YG5 (Nb-20Si-5Mo-3W), and YG8 (Nb-20Si-5Hf-5Mo-3W) [31] (respectively 8.38, 8.63, and 8.67 g/cm^3^) and (b) the target density (ρ = 9 g/cm^3^) of Nb-silicide-based alloys with RM additions and with strength of 450 MPa at 1500 °C [18]. However, the densities of JZ1 and JZ2 were higher than those (i) of the Al and/or Cr containing JG series of alloys with/out Mo, Hf, or Sn (6.55 ≤ ρ ≤ 7.68 g/cm^3^), (ii) of the Al and/or Cr containing KZ series of alloys with/out Ta (6.55 ≤ ρ ≤ 7.15 g/cm^3^), (iii) of the Ge containing ZF series of alloys with/out Al and/or Cr or Hf additions (6.14 ≤ ρ ≤ 7.99 g/cm^3^).

### 5.2. Macrosegregation

Macrosegregation of Si (MACSi) existed in both alloys. The latter is defined as MACSi = C_max_^Si^ − C_min_^Si^ where C_max_^Si^ and C _min_^Si^, respectively are the maximum and minimum Si concentrations in the cast alloy [26,27]. The MACSi values were 5.6 and 4.9 at.% for the alloys JZ1 and KZ2, respectively. In Nb-silicide-based alloys, the increase of MACSi has been correlated with the increase and decrease of specific alloy parameters [26,27] and such trends have been confirmed in Nb-silicide-based alloys with a wide range of alloying additions and concentrations [26,27]. To our knowledge, there is no data about the macrosegregation of Si in Nb-silicide-based alloys with Ta or W, and Ge or Sn as alloying elements that would allow studying the parameter trends for the alloys JZ1 and JZ2.

### 5.3. Microstructures

In both alloys the primary phase was the βNb_5_Si_3_. In the alloy JZ1, as the primary silicide formed the surrounding melt became rich in Sn, Ta, Ti, and W owing to the partitioning of these solutes. In this melt the Nb_ss_ and the Nb_ss_ + βNb_5_Si_3_ eutectic formed, and Nb_ss_ halos formed around the primary βNb_5_Si_3_ grains. Some Nb_ss_ halos surrounded the primary βNb_5_Si_3_ completely (Figure 2a). Halo formation results from the competitive growth of eutectic phases in the solidification of a melt of off-eutectic composition. In the eutectic in JZ1, the Si + Ge + Sn content was 20.7 at.%, which is close to the composition of the metastable Nb_ss_ + βNb_5_Si_3_ eutectic in the Nb-Si binary [32] and falls within the range of Nb_ss_ + βNb_5_Si_3_ eutectic compositions in Nb-silicide-based alloys [13] and references within. 

Both βNb_5_Si_3_ and αNb_5_Si_3_ were present in JZ1-AC (Figure 1a). The αNb_5_Si_3_ can form on cooling from the βNb_5_Si_3_ or from the Nb_3_Si via a eutectoid transformation [31,33]. Solute additions (i) stabilize the βNb_5_Si_3_ [13,31], (ii) promote the βNb_5_Si_3_ → αNb_5_Si_3_ transformation, and (iii) stabilize or destabilize the tetragonal Nb_3_Si [13]. We can understand these effects by considering the crystal structures of M_5_Si_3_ and M_3_Si silicides and the stability of the latter in Nb-Si-TM/RM ternaries. 

In the top and bulk of JZ1-AC, the Si concentration was in the range 20.4 to 24.4 at.% and 21.2 to 23 at.%, respectively (Table 1). Thus, in both cases and according to the Nb-Si binary phase diagram the primary phase should be the βNb_5_Si_3_. The Nb_3_Si would form via L + βNb_5_Si_3_ → Nb_3_Si and then αNb_5_Si_3_ from βNb_5_Si_3_ + Nb_3_Si → αNb_5_Si_3_ and Nb_3_Si → Nb_ss_ + αNb_5_Si_3_. In the top and bulk of the button of JZ1-AC, no evidence was found for Nb_3_Si, and microstructures resulting from these phase transformations.

The solutes Ta and Ti form tetragonal M_3_Si silicides that have the same crystal structure as Nb_3_Si (tP32, prototype Ti_3_P) and that are stable at room temperature, and thus would be expected to encourage the formation of the latter. The solutes Hf and W do not form M_3_Si silicides and thus would be expected to have the opposite effect. Furthermore, Sn suppresses the tetragonal Nb_3_Si on its own or with Ti [26], or Hf [34], meaning that Sn has a very strong effect on the stability of the tetragonal Nb_3_Si. Germanium could also have the same effect, which was compromised when it was added to a Nb-silicide-based alloy with Ti. The solute elements Ge, Ta, W form tetragonal 5-3 silicides that have the same crystal structure as the βNb_5_Si_3_ (tI32, D8_m_, prototype W_5_Si_3_) [35], and thus would be expected to stabilize the latter but Sn promotes the βNb_5_Si_3_ → αNb_5_Si_3_ transformation on its own or with Ti. The solute elements Sn and W promote the Nb_ss_ + Nb_5_Si_3_ eutectic in ternary Nb-Si-X alloys [20]. In the case of W, the Nb_ss_ + Nb_3_Si eutectic is suppressed and replaced by the Nb_ss_ + βNb_5_Si_3_ eutectic when W ≥ 3 at.% in the ternary alloy [20]. 

It is suggested that in the top and bulk of JZ1-AC the solidification path was L → L + βNb_5_Si_3_ → βNb_5_Si_3_ + (Nb_ss_ + βNb_5_Si_3_)_eutectic_ with the tetragonal Nb_3_Si suppressed for the reasons discussed above. The formation of αNb_5_Si_3_ in these parts of the button occurred during solid state cooling and was promoted by Sn [31,34]. 

Disagreements about the Si content of different phases, and temperatures of phase equilibria in the Nb-Si binary system have been highlighted by David et al. [36]. For example, the temperature and composition of the liquid (i) for the L → Nb + Nb_3_Si eutectic transformation have been reported to be respectively in the ranges 1912 to 1938 °C and 15.3 to 18.7 at.% Si and (ii) for the L + βNb_5_Si_3_ → Nb_3_Si peritectic transformation to be respectively in the ranges 1968 to 1997 °C and 17 to 21.1 at.% Si. In the bottom of the button of JZ1-AC the Si concentration was in the range 19 to 24.6 at.%. Considering the available Nb-Si binary phase diagrams, for Si concentrations of the melt between the compositions of the liquid for the above eutectic and peritectic transformations the solidification path should be L → L + Nb_3_Si → Nb_3_Si + (Nb_ss_ + Nb_3_Si)_eutectic_ → (Nb_ss_ + Nb_3_Si)_eutectic_ + (Nb_ss_ + αNb_5_Si_3_)_eutectoid_ and for Si concentrations exceeding the composition of the liquid for the above peritectic transformation the solidification path should be L + βNb_5_Si_3_ → Nb_3_Si and then αNb_5_Si_3_ should form from the peritectoid transformation βNb_5_Si_3_ + Nb_3_Si → αNb_5_Si_3_ and the eutectoid transformation Nb_3_Si → Nb_ss_ + αNb_5_Si_3_. 

In the microstructure in the bottom of the button of JZ1-AC there was no evidence of either of the above solidification paths. Instead, the microstructure was (a) either a mixture of coarse Nb_ss_ and Nb_5_Si_3_ (Figure 2c) with a transition to the βNb_5_Si_3_ + (Nb_ss_ + βNb_5_Si_3_)_eutectic_ bulk microstructure (Figure 2b), or (b) a mixture of Nb_ss_, Nb_5_Si_3_, and Nb_3_Si (Figure 2d,e,f) (which was not observed in JZ2-AC), with a transition to the bulk microstructure, i.e., βNb_5_Si_3_ + (Nb_ss_ + βNb_5_Si_3_)_eutectic_ (Figure 2d, notice scarce Nb_ss_ halos around the Nb_5_Si_3_ in the βNb_5_Si_3_ + (Nb_ss_ + βNb_5_Si_3_)_eutectic_ microstructure in this figure) and there was evidence of the transformation Nb_3_Si → (Nb_ss_ + αNb_5_Si_3_)_eutectoid_, see Figure 2f. The dominant microstructure was the mixture of coarse Nb_ss_ and Nb_5_Si_3_ followed by a transition to the βNb_5_Si_3_ + (Nb_ss_ + βNb_5_Si_3_)_eutectic_ bulk microstructure, see Figure 2b,c.

In as-cast Nb-Si-Ge ternary alloys the microstructure that formed in contact with the water-cooled crucible consisted of a mixture of Nb_ss_ and Nb_5_Si_3_, like the microstructure in Figure 2c, and there was a transition of the latter (anomalous eutectic) to regular eutectic to Nb_5_Si_3_ + (Nb_ss_ + Nb_5_Si_3_)_eutectic_ (as shown in Figure 2b) further away from the water-cooled side toward the bulk of the button. The “width” of the former zone (mixture of Nb_ss_ and Nb_5_Si_3_) increased with Ge content in the alloy. The microstructures seen in Figure 2c,d were attributed to the addition of Ge in the alloy JZ1. 

In cast Nb-silicide-based alloys prepared using arc melting, different zones and/or transitions are observed in the microstructures that are formed from the undercooled melt that was in contact with the water-cooled copper crucible [26]. A eutectic that exhibits a transition from anomalous to regular eutectic with decreasing melt undercooling (ΔT_melt_) usually consists of a solid solution with an intermetallic. The coarse microstructure in the bottom of JZ1-AC (Figure 2c) consisted of the solid solution Nb_ss_ and the Nb_5_Si_3_ intermetallic. These phases have different crystal structures and the Nb_5_Si_3_ has higher entropy of fusion (ΔS_f_ = 14.55 J/mol K) than Nb_ss_ (ΔS_f_ = 9.45 J/mol K). High ΔS_f_ signifies flat (facetted, sharp) S/L interface and thus kinetic undercooling is required for growth of the interface. In other words, the Nb_5_Si_3_ would require kinetic undercooling to grow. In unconstrained solidification of eutectic melts (as is the case of solidification in a water-cooled copper crucible) a transition from purely anomalous eutectic to a mixture of regular and anomalous eutectic to regular eutectic has been reported with decreasing melt undercooling [37]. 

It is suggested that in the bottom of the button of JZ1-AC the undercooling of the melt was ΔT_melt_ > ΔT_critical_, with ΔT_critical_ ≈ 0.25ΔT_max_, where ΔT_critical_ and ΔT_max_ are respectively the critical undercooling for the formation of anomalous eutectic and the maximum undercooling achievable in the eutectic system [38]. The condition ΔT_melt_ > ΔT_critical_ meant different growth rates for the Nb_ss_ and Nb_5_Si_3_, with the former growing faster, owing to its low ΔS_f_, which signifies diffuse (rough) S/L interface than the latter with higher ΔS_f_ (see above). This caused decoupled growth of the two phases of the eutectic and led to the anomalous microstructure in the bottom. As the latter grew the released latent heat of solidification increased the temperature of the melt ahead of the S/L interface which led to lower growth rates and eventually coupled growth between the two phases became possible and resulted in the formation of the regular eutectic.

A microstructure consisting of three phases (Nb_ss_, Nb_3_Si, Nb_5_Si_3_) was seen in parts of the bottom of the JZ1-AC, see Figure 2d,e, where the volume fraction of the Nb_3_Si was higher than the other two phases. This microstructure could be a ternary eutectic consisting of the aforementioned phases. In parts of this three phase microstructure the Nb_3_Si had transformed, as shown in Figure 2f, and the morphological characteristics of the transformation are typical of the eutectoid transformation Nb_3_Si → Nb_ss_ + αNb_5_Si_3_. Some of the αNb_5_Si_3_ that was detected by XRD must be attributed to this phase transformation.

In the Nb-Si binary system the Nb_3_Si can form as the primary phase when the Si content of the melt is greater than the eutectic composition (see above and [36]). In the bottom of the button of JZ1-AC where the Nb_3_Si was observed, the lowest Si concentration was 19 at.% (Table 1). However, the Nb_3_Si did not form in the bottom of JZ2-AC where the lowest Si concentration was 18.8 at.%, essentially the same with JZ1 (Table 3). This difference was attributed to the higher Sn concentration in the alloy JZ2, compared with the alloy JZ1, and the strong destabilizing effect of Sn on the tetragonal Nb_3_Si (see above). The Nb_3_Si formed in the bottom of JZ1 because of the Si content of the melt that solidified in contact with the water-cooled crucible (see above) and because the chemical composition of the microstructure shown in Figure 2e,f was essentially the same as that of the bottom of JZ1-AC but with Ge/Sn = 1 (see Section 4.1) instead of Ge/Sn = 1.6 (Table 1). This would suggest that at low Sn contents in the alloy, the Ge, which can destabilize the tetragonal Nb_3_Si but not as effectively as Sn, is required to “help” the Sn suppress the tetragonal Nb_3_Si. 

The Figure 2b shows a transition from a Nb_ss_ and Nb_5_Si_3_ coarse microstructure to the Nb_5_Si_3_ + (Nb_ss_ + Nb_5_Si_3_)_eutectic_ microstructure, in other words it shows that the latter “grew” from the former. It is suggested that in the parts of the bottom of the button where the tetragonal Nb_3_Si had formed, this transition occurred when the growth of Nb_3_Si was destabilized by high Sn concentration in the melt ahead of the ternary eutectic/melt interface (Sn has negligible solubility in Nb_3_Si and low solubility in Nb_5_Si_3_, see Table 1, thus the solidification of the ternary was accompanied by partitioning of Sn in the melt) so that only Nb_ss_ and Nb_5_Si_3_ could form, and then under the condition ΔT_melt_ > ΔT_critical_ the two phases (Nb_ss_ and Nb_5_Si_3_) grew, as discussed above. 

The microstructure in the top and bulk of the button of JZ2-AC was formed with the same solidification path as discussed above for the top and bulk of JZ1-AC. The finer scale of the eutectic in the top and bulk of the alloy JZ2 compared with the alloy JZ1 (Figure 2a and Figure 5a) was attributed to the higher Ge content in the alloy JZ2 and the higher Ge/Sn ratio in the top and bulk. Germanium refines the eutectic microstructure in ternary Nb-Si-Ge alloys. The average Si + Ge + Sn content of the eutectic was 23.3 at.%. The latter is in the range of the compositions of eutectics with Nb_ss_ and βNb_5_Si_3_ that are formed in Nb-silicide-based alloys [13] and references within. 

The A15 compound was observed only in the bottom of JZ2-AC where the melt was richer in Sn compared with the bulk and top (Table 3). Researchers have suggested (i) that formation of A15-Nb_3_Sn in Nb-silicide-based alloys depends strongly on their Sn content and (ii) that a minimum Sn concentration is required for the Nb_3_Sn to be stabilized in the microstructure [3,26,39]. This is supported by the results for the alloys JZ1 and JZ2.

The microstructure that formed in the bottom of the button of JZ2-AC was slightly different compared with JZ1-AC. Indeed, the phases that formed in JZ2-AC were the Nb_ss_, A15, and Nb_5_Si_3_ and there was no Nb_5_Si_3_ + Nb_ss_ eutectic and no Ti-rich Nb_ss_ and Ti-rich Nb_5_Si_3_ (bottom left hand corner in Figure 5d). The entropies of fusion of the Nb_ss_, A15-Nb_3_Sn, and Nb_5_Si_3_ are 9.45, 11.6, and 14.55 J/mol K, respectively. Thus, the growth of the former two would be expected to be easier than that of the Nb_5_Si_3_, which would require kinetic undercooling (see above). 

Niobium has the bcc structure, which is also the structure of the Nb solid solution in Nb-silicide-based alloys [14]. Niobium can form a metastable A15-Nb_3_Nb structure with 0.5246 nm lattice parameter [40]. The lattice parameter of the stoichiometric A15-Nb_3_Sn is 0.529 nm [41]. In the A15 structure the Sn atoms form a bcc lattice, each face of the cube is bisected by orthogonal Nb chains and the distance between the Nb atoms is 0.265 nm. In the A15-Nb_3_Nb structure the distance between the Nb atoms is 0.262 nm. In both cases these distances are lower than the shortest distance between Nb atoms in bcc Nb, which is 0.286 nm (the Nb lattice parameter is 0.330 nm) [42]. Both Ta and Ti occupy Nb sites in the A15-Nb_3_Sn lattice [43] and as their concentrations increase, the stability of the cubic phase increases. 

The compositions of the phases and the eutectic in Table 3 and the fact that no Ti-rich Nb_ss_ and Ti-rich Nb_5_Si_3_ were observed in the microstructure that formed in the bottom of the button of JZ2-AC (bottom left hand corner in Figure 5d) show that the partitioning of solutes in Nb_5_Si_3_ created favorable concentrations for the Ge, Si, Sn, Ta, Ti, W in the eutectic, but the partitioning of solutes in the solid solution and A15 created favorable concentrations for Ge and Si, and Ge, Si, and Ta, respectively.

In the undercooled melt of JZ2, near the water-cooled crucible the Nb_5_Si_3_ formed first (owing to its higher melting temperature) and then the Nb_ss_, the growth of which was easier because of its lower entropy of fusion (see above). As solutes partitioned to the melt the formation of A15-Nb_3_Sn next to the solid solution became possible because of crystallographic reasons and the availability of Ge and Si that also form A15 compounds [35]. Thus, growth of the three phases became possible but the eutectic did not form because the melt could not reach the eutectic composition owing to the growth of the three phases separately. However, as the latter grew the melt undercooling decreased making it possible for the Ti-rich Nb_ss_ and Ti-rich Nb_5_Si_3_ to form. Formation of the latter two phases made less Ge, Si and Ti available for the needs of the A15-Nb_3_Sn and thus its formation/growth was suppressed and this marked the start of the transition from the microstructure in the bottom of JZ2-AC to that in the bulk, with coupled growth of Nb_ss_ and Nb_5_Si_3_ resulting in the eutectic between these two phases (Figure 5b,d).

The dependence (sensitivity) of the microstructures that formed in the cast alloys JZ1 and JZ2 on solidification conditions was discussed above and is common in Nb-silicide-based alloys that are produced using arc melting, as demonstrated in [25,26,31,34]. The importance of macro-segregation and solidification conditions for the cast microstructures and properties of arc melted RCCAs have been highlighted in [8]. The lack of research on the solidification of RCCAs is noticeable. 

In the microstructures of the heat-treated alloys JZ1 and JZ2, the Nb_ss_, and βNb_5_Si_3_ and αNb_5_Si_3_ were present with Ti-rich areas being present only in the Nb_5_Si_3_ silicide where the Ti+Hf content was not high enough to stabilize the hexagonal γNb_5_Si_3_. This suggests that the solid solution and the tetragonal Nb_5_Si_3_ silicides are stable in both alloys. The presence of both forms of the tetragonal Nb_5_Si_3_ is attributed to the βNb_5_Si_3_ stabilizers Ta, W, and Ge “controlling” the effect of Sn that promotes the βNb_5_Si_3_→αNb_5_Si_3_ transformation. In JZ2-HT, the A15 compound was stabilized owing to the higher Sn content in the alloy compared with JZ1 as well as the presence of Ge and Si that stabilizes this structure. Based on the results for the alloy CM1 (=Nb-8.3Ti-21.1Si-5.4Mo-4W-0.7Hf) in [31], it is suggested that the subgrains (Figure 3b) formed in the αNb_5_Si_3_ grains in JZ2-HT.

In both alloys the eutectic microstructures were not stable after the heat treatment. This was not observed in the alloys Nb-17.4Si-5.3Hf-3.4Ta-2Mo and Nb-19.7Si-2W-4.5Mo [30] with no Ti addition where lamellar microstructures consisting of Nb_ss_ and Nb_5_Si_3_ were present after the heat treatment. This difference was attributed (i) to the synergy of the refractory metals with Ge and Sn and (ii) to the higher homologous heat treatment temperatures for the alloys JZ1 and JZ2 that were about 0.74 and 0.76, respectively, compared with the homologous temperature of about 0.69 for the two aforementioned alloys.

The partitioning of Ti, and Ta and W in the Nb_ss_ and Nb_5_Si_3_ in the alloys JZ1 and JZ2 was similar to that of Ti, and Mo and W [31], and Ti and Ta [44]. Indeed, as the concentration of Ti in these phases increased, the RM content decreased. 

### 5.4. Oxidation

#### 5.4.1. Oxidation at 800 °C

At 800 °C, both alloys followed linear oxidation kinetics, did not form protective scales, and suffered from the spallation of their scales, parts of which disintegrated into powder. The latter revealed their vulnerability to pesting (Figure 7a,b) and drew attention to the need for future research to find out if additional alloying could suppress it. The values of their rate constants were higher than those of the MASC alloy (Nb-25Ti-16Si-8Hf-2Al-2Cr) with/out Sn addition [24], similar to those of Al and Cr containing Nb-silicide-based alloys with/out Hf addition but without Sn or Ge and higher than those of Al and/or Cr containing Nb-silicide-based alloys with 2 or 5 at.% Sn [26]. Their oxidation was worse than that of the Al and/or Cr containing Nb-silicide-based alloys with Ge in [25], and the alloy OHS1 (Nb-24Ti-18Si-5Al-5Cr-5Ge-5Sn [27]) that followed parabolic oxidation kinetics at the same temperature. The weight gains of both alloys were higher than those of Nb-silicide-based alloys with Ge [25] or Sn [24,26], the alloy OHS1 [27] and Al and Cr containing alloys with/out Hf addition.

Pest oxidation of intermetallic compounds and alloys occurs in the range 600 to 900 °C. Pesting has been attributed to the rapid diffusion of oxygen along the grain boundaries that causes internal oxidation. The oxide(s) that are formed induce internal stresses that cause disintegration along the grain boundaries [45]. The vol.% of the Nb_ss_ is critical for the oxidation of Nb-silicide-based alloys [25,28]. In Nb-silicide-based alloys, the Nb_5_Si_3_ is cracked below the substrate/scale interface with the cracks running parallel to the scale/substrate interface. This phenomenon was attributed to the low fracture toughness of the Nb_5_Si_3_ (about 3 MPa m^0.5^ for the unalloyed silicide which increases to about 10 MPa m^0.5^ for Ti alloyed silicide with ≈ 30 at.% Ti [29]) and the stresses arising from the oxidation of the Nb_ss_ [21,24,26]. Control of the vol.% of the Nb_ss_ and the diffusion of oxygen toward the bulk of the alloy are critical for the pest oxidation of Nb-silicide-based alloys. The increase of the Sn and Ge concentrations in JZ2 and their synergy with Hf, Si, Ta, Ti, and W had an effect only on the vol.% of Nb_ss_, which was lower than that of JZ1 (Table 2).

The vol.% of the Nb_ss_ in the alloys JZ1 and JZ2 respectively were 46.5 % and 34.5 %. The alloy Nb-18Si-5Sn (NV9) suffered from complete disintegration to powders (pesting) at 800 °C and that the alloy Nb-24Ti-18Si-5Sn (NV6) did not pest but instead formed a thin scale that cracked along all the edges of the cube shaped specimen. The alloy Nb-24Ti-18Si-5Ge (ZF3) also suffered from complete disintegration to powders at 800 °C [25]. The results for the alloy NV6, NV9, and ZF3 would suggest that the synergy of 5 at.% Sn with 24 at.% Ti is more effective than that of 5 at% Ge with 24 at% Ti regarding the control of pest oxidation at 800 °C.

The alloys JZ1 and JZ2 had lower Ge and Sn concentrations and the same but lower Ti concentrations than the alloys studied by Vellios and Tsakiropoulos (see [13]) and Li and Tsakiropoulos [25] and references within and did not suffer from complete disintegration to powders, instead their scales spalled off and scale fragments disintegrated into powder. This suggests (i) that the simultaneous presence of Ge and Sn in the alloys “controlled and improved” the effect of Ge and (ii) that the synergy of these two elements with Hf, Ta, Ti, and W was also beneficial regarding control of pest oxidation. The oxidation resistance of the alloy JZ2 at 800 °C was slightly improved, most likely because of its lower vol.% of the Nb_ss_ and higher Sn+Ge content, compared with the alloy JZ1. The results of this work and the literature suggest that additional research should aim to find out whether the oxidation of Nb-silicide-based alloys with Ta and W additions in the pest regime could be improved with simultaneous addition of Sn and Ge with Al and Cr.

#### 5.4.2. Oxidation at 1200 °C

The results for the two alloys and in particular the improved adhesion of the scale of JZ2 showed that their oxidation was similar or better than that of some Nb-silicide-based alloys with Al and Cr additions. At the early stages, the oxidation of the alloys JZ1 and JZ2 was parabolic, and then changed to linear (Table 4). Their weight gains after 50 h were similar to those of the MASC alloy with/out Sn addition [24] (in [24] the oxidation at 1200 °C was studied up to 50 h) and their k_l_ values were the same as those of MASC [24]. Their weight gains were higher than those of Nb-silicide-based alloys with/out Al or Cr and with 2 or 5 at.% Sn [26] and references within, and the alloy OHS1 [27]. Compared with the latter, the linear oxidation rate constants of JZ1 and JZ2 were similar but the parabolic rate constant was higher. Their rate constants were both slightly higher than those of the alloys with 2 at.% Sn [26] and their k_l_ values were similar to those of the alloys ZX6 (Nb-24Ti-18Si-5Al-5Sn) and ZX8 (Nb-24Ti-18Si-5Al-5Cr-5Sn) [26] and references within, of which the latter did not follow parabolic kinetics in the early stages of oxidation. Compared with the Ge containing alloys in [25], the weight gains of JZ1 and JZ2 were higher, and the k_l_ and k_p_ rate constants of the alloy JZ2 were similar to those of Nb-24Ti-18Si-5Cr-5Ge. 

The scale formed on JZ1 spalled off (Figure 7c), as was the case for (a) the MASC alloy with/out Sn addition [24], (b) the alloys JG3 (Nb-24Ti-18Si-5Al-5Cr-2Mo) and JG4 (Nb-24Ti-18Si-5Al-5Cr-5Hf-2Mo, which did not follow parabolic kinetics), (c) the alloys with 2 at.% Sn in [26], (d) the alloys with 5 at.% Ge in [25], and (e) the alloys with 5 at.% Sn in [26]. Partial spallation and improved scale adhesion was exhibited by the alloy JZ2 (Figure 7), similar to the alloys ZX6 (Nb-24Ti-18Si-5Al-5Sn) [26] and ZF9 (Nb-24Ti-18Si-5Al-5Cr-5Hf-5Ge) [25]. Thus, the synergy of Sn and Ge with Ta and W had the same effect regarding scale adhesion as 5Al+5Sn in ZX6 and 5Al+5Cr+5Ge+5Hf in ZF9, but was not as effective as that of 5Sn+5Ge with 5Al+5Cr in OHS1 [27] and 5Sn with 5Al, 5Cr, 5Hf, and 2Mo in JG6 (Nb-24Ti-18Si-5Al-5Cr-5Hf-5Sn-2Mo) where the vol.% of the Nb_ss_ in the cast alloy was very low and the solid solution was not stable (note that the Sn rich Nb_ss_ that was reported in JG6-HT [46] actually was the A15-Nb_3_Sn compound). Could simultaneous Al and Cr additions to the “base” alloy JZ2 improve oxidation at 1200 °C? 

In the alloy JZ2 the scale was free of Sn or Ge as was the case in the alloys with 2 and 5 at.% Sn [26], and with 5 at.% Ge [25] and the alloy OHS1 with 5Sn+5Ge [27]. The presence of Nb and Ti rich oxides and silica in the scale is in agreement with [26,46]. In Nb-silicide-based alloys with Al and/or Cr and with Sn and/or Ge additions, Sn-rich and/or Ge-rich intermetallics formed in the substrate in the diffusion zone just below the scale/substrate interface [24,25,26,27]. In the alloy JZ2, Ge and Sn segregated in the substrate just below the scale/substrate interface where Sn rich and Ge rich areas formed (Figure 8 and Figure 9) as well as Sn and Ge containing intermetallic compounds (Table 5). The diffusion zone consisted of two parts, owing to differences in microstructure (Figure 8).

Segregation of Sn and Ge in the substrate just below the scale/substrate interface has been reported before, but always for Nb-silicide-based alloys that contained Al and/or Cr, with/out Hf [20,22,23,25]. A distinctive feature of JZ2, which has not been observed before, was the presence “next to each other” of separate Sn rich or Ge rich areas at the top of the diffusion zone 1 near its interface with the scale (Figure 8a,c and Figure 9). Table 6 shows the alloying additions in JZ1 and JZ2 that are predicted to segregate to the surfaces of binary Nb-X alloys [26] and references within. According to theory A, the solute with lower heat of sublimation should segregate to the surface. In accordance with theory B, the larger the solute atom relative to the solvent the higher the degree of surface segregation. As per theory C, the surface segregation is related with the partitioning of solute in the melt and surface segregation should occur when, owing to distribution (partitioning) of solute, the melt is richer in solute than the solid. Finally, as stated by theory D, the element with the lower surface energy segregates. None of the theories A to D predicts surface segregation of W or Ta. The predictions of theories A to D are supported by the analysis data (Table 5) and X-ray elemental maps (Figure 9). 

The microstructure of the diffusion zone 2 was essentially the same as in the bulk. Titanium oxide was observed either inside the Nb_ss_ and A15 compound or at their interface. This confirmed that in JZ2 both these phases were more susceptible to contamination by oxygen than the Nb_5_Si_3_ at 1200 °C. Contamination of the microstructure of JZ2 by oxygen had occurred to the depth of about 200 μm, i.e., deeper than that in the alloy OHS1 (=Nb-24Ti-18Si-5Al-5Cr-5Ge-5Sn [27]), which was about 100 μm. This would suggest that the synergy of Ta and W with Ge and Sn was not as effective as that of the latter elements with Al and Cr, even in the presence of the oxygen scavenging Hf in JZ2. 

Contamination by oxygen of phases below the scale and sometimes in the bulk of Nb-silicide-based alloys also has been reported in [26,27]. The presence of Ge containing compounds, with the exception of NbGe_2_, and A15 intermetallic below the scale (Table 5) is consistent with the data in [24,26,27]. However, the absence of other Sn intermetallics (NbSn_2_, (Ti,Nb)_6_Sn_5_, and TM_5_Sn_2_Si), which were observed in [26,27], also should be noted. These observations would suggest (a) that the presence of Al and/or Cr is not essential for the formation of Ge and Sn containing intermetallic phases below the scale, and (b) that the alloying with RMs (synergy of Ta with W in this study) (i) suppresses the formation (stability ?) of certain Ge and Sn rich compounds below the scale, (ii) affects solute partitioning in Nb_5_Si_3_ below the scale (compare the data for Nb_5_(Si_1-x_Ge_x_)_3_ and W-rich Nb_5_(Si_1-x_Ge_x_)_3_ with that for Nb_5_Si_3_ and Ti-rich Nb_5_Si_3_ in Table 5) and promotes the formation (stability?) of NbGe_2_ below the scale. Further research is necessary to clarify (b).

### 5.5. Comparisons of Experimental Data with NICE 

The alloy design methodology NICE [13] was used to calculate (predict) the macrosegregation of Si (MACSi), the composition of the Nb_ss_ and Nb_5_Si_3_, the vol.% of Nb_ss_, and the weight gains per unit area (ΔW/A) after isothermal oxidation at 800 and 1200 °C. The latter were compared with the experimental data. The creep rate of the alloys at 1200 °C and 170 MPa was also calculated using NICE. 

NICE slightly under and over-estimated the MACSi values, respectively in the alloys JZ1 and JZ2, which, based on their actual compositions, were calculated as being equal to 4.8 at.% and 5.2 at.%.

The Si concentration in the Nb_ss_ in JZ1-AC, JZ1-HT, and JZ2-HT respectively was 4.4, 3.8, and 3.5 at.%. These Si contents were close to those reported for the Nb_ss_ in the Al, Cr, and Ta containing alloys KZ6 (=Nb-24Ti-18Si-6Ta-5Al-5Cr) and KZ8 (=Nb-24Ti-18Si-6Ta-4Al-8Cr) in the as-cast and heat-treated conditions [44] and the “normal” Nb_ss_ in the Ta-containing and Al-, Cr-, and Ti -free alloy YG4-AC (=Nb-18Si-5Hf-3Ta-2Mo), and significantly higher, particularly for the heat-treated condition, than the values reported for the Nb solid solutions in Nb-silicide-based alloys without Ta addition, or with Ge addition [25] or with Sn addition [26,34]. 

The chemical compositions of the Nb_ss_ in the as-cast condition that were calculated using the actual compositions of the cast alloys and the relationships in NICE that link the concentration of a solute element in the alloy and its solid solution were 65.35Nb-14.15Ti-5.5Si-8.4Ta-4.1W-0.6Hf-1.4Sn-0.5Ge and 63.4Nb-13.3Ti-4Si-9Ta-4.8W-0.76Hf-3.3Sn-1.5Ge, respectively for the alloys JZ1 and JZ2. The Ta concentration was calculated using data for Mo in NICE, because there are not enough experimental data available for Ta containing Nb-silicide-based alloys. The values of the parameters δ and Δχ of the calculated Nb_ss_ respectively were 5.091 and 0.179, and 5.087 and 0.1946 for the alloys JZ1 and JZ2, and were consistent with [14,44]. The calculated compositions of the solid solutions were in very good agreement with the data in the Table 1 and Table 3. The values of the parameters Δχ and δ calculated for the actual compositions of the “normal” and Ti-rich Nb_ss_ in the alloys JZ1 and JZ2 (Table 1 and Table 3), respectively were 0.182 and 0.2109, and 5.858 and 5.363, and were consistent with [14]. 

The calculated values of the vol.% Nb_ss_ using NICE were 44.1% and 34.8%, respectively for the alloys JZ1 and JZ2, in very good agreement with the measured vol.% Nb_ss_ in the bulk of both the as-cast alloys (Table 2).

In the Nb_5_Si_3_ database in NICE the data for RM additions in tetragonal Nb_5_Si_3_ is limited. This lack of data hampered the calculation of the concentrations of Ta and W in the latter silicide. Calculation of the concentrations of the other elements in the alloyed Nb_5_Si_3_ was possible. For example, using the actual JZ2-AC alloy composition, the calculated composition of the Nb_5_Si_3_ was 49.9Nb-10Ti-5.8Ge-0.95Sn-0.7Hf-32.65Si. 

The average weight gains of the alloys JZ1 and JZ2 at 800 °C and 1200 °C that were calculated using NICE, respectively were 17 and 12 mg/cm^2^, and 77 and 54 mg/cm^2^. Compared with the data in Table 4, NICE underestimated the ΔW/A values for both alloys and temperatures. The better oxidation behavior of JZ2 compared with JZ1 correlated well with the decrease in VEC and increase in δ parameter values (see next section), as required by NICE [13,25,26,27].

#### Creep

The values of the parameters VEC, Δχ, and δ and the ratio Nb/(Ti+Hf) that were calculated for the actual compositions of the cast alloys JZ1 and JZ2, respectively were 4.642, 0.1745, 8.93, and 4.41, and 4.562, 0.1859, 9.14 and 3.95. Both alloys met the constraint (a) (see Section 2). The calculated creep rates at 1200 °C and 170 MPa using NICE and the values of the above parameters and ratio were in the ranges 1.4 × 10^−6^ s^−1^ to 1.1 × 10^−7^ s^−1^, and 1.5 × 10^−6^ s^−1^ to 1.8 × 10^−7^ s^−1^, with average creep rates 7 × 10^−7^ s^−1^ and 9.6 × 10^−7^ s^−1^, respectively for the alloys JZ1 and JZ2. These rates were lower than that of the Ni-based superalloy CMSX-4 for the same conditions (5.6 × 10^−5^ s^−1^, see Section 2) but higher than the creep rate of 10^−7^ s^−1^ that is the criterion in NICE to predict whether it is likely for a designed (selected) alloy to meet the creep goal (see Section 2 and [13]). 

## 6. Summary 

Two Nb-silicide-based alloys with nominal compositions Nb-12Ti-18Si-6Ta-2.5W-1Hf-2Sn-2Ge (JZ1) and Nb-12Ti-18Si-6Ta-2.5W-1Hf-5Sn-5Ge (JZ2) were designed using the alloy design methodology NICE. All the research objectives that were discussed in the introduction of this paper were met. The cast microstructures of both alloys were sensitive to solidification conditions. There was macrosegregation of Si in JZ1 and JZ2. In both alloys the βNb_5_Si_3_ was the primary phase and the Nb_ss_ was stable. The A15-Nb_3_X (X = Ge,Si,Sn) was stable only in JZ2. The Nb_3_Si and Nb_ss_ + βNb_5_Si_3_ eutectic that formed only in JZ1 were not stable. Also the Nb_ss_ + βNb_5_Si_3_ eutectic was not stable in JZ2. At 800 °C both alloys followed linear oxidation kinetics and were vulnerable to pesting. At 1200 °C both alloys exhibited parabolic oxidation kinetics in the early stages that was followed by linear kinetics. The adhesion of the scale on JZ2 was better. The microstructure of JZ2 was contaminated by oxygen to a depth of about 200 μm, and in the substrate below the scale there was presence of NbGe_2_, Nb_5_(Si_1-x_Ge_x_)_3_, W-rich Nb_5_(Si_1-x_Ge_x_)_3_, and A15-Nb_3_X compounds. The better oxidation behavior of JZ2 compared with JZ1 correlated well with the decrease in VEC and increase in δ parameter values, in agreement with NICE. The experimental data for Si macrosegregation, vol.% Nb_ss_, chemical composition of Nb_ss_ and Nb_5_Si_3_, weight gains at 800 and 1200 °C was compared with the calculations (predictions) of NICE. The agreement was very good. The calculated creep rates of both alloys at 1200 °C and 170 MPa were lower than that of the Ni-based superalloy CMSX-4 for the same conditions but higher than 10^−7^ s^−1^. 

## Figures and Tables

**Figure 10:**
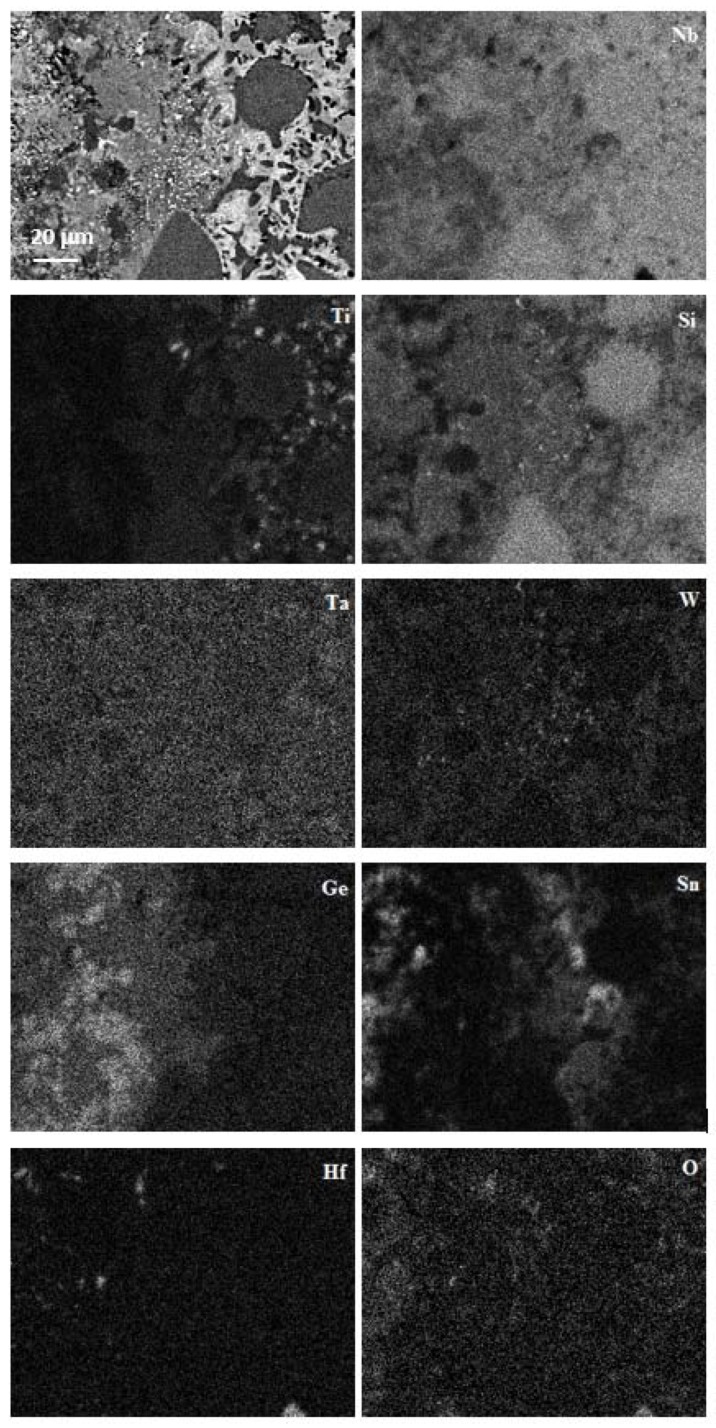
BSE image and X-ray maps of the rectangular region in Figure 8d.

**Table 1:** The analysis data (at.%) for the alloy JZ1.

Area Phase	Nb	Ti	Si	Ta	W	Sn	Ge	Hf
As-cast								
Top	55.1 ± 0.8 54.0–55.9	11.9 ± 0.6 11.3–12.7	22.3 ± 1.7 20.4–24.4	5.4 ± 0.3 5.2–5.8	1.6 ± 0.3 1.4–2.0	1.1 ± 0.2 0.9–1.3	1.8 ± 0.1 1.7–2.0	0.8 ± 0.1 0.7–0.9
Bulk	55.1 ± 0.9 53.5–56.0	11.4 ± 0.2 11.1–11.6	22.9 ± 1.6 21.2–23.0	5.1 ± 0.4 4.6–5.7	1.6 ± 0.3 1.3–1.9	1.2 ± 0.1 1.1–1.3	1.8 ± 0.2 1.6–2.1	0.9 ± 0.2 0.8–1.1
Bottom	55.4 ± 2.0 53.5–57.6	11.4 ± 0.2 11.3–11.7	22.3 ± 2.6 19.0–24.6	5.5 ± 0.4 5.1–6.2	1.7 ± 0.4 1.3–2.3	1.1 ± 0.2 0.9–1.3	1.8 ± 0.2 1.6–2.0	0.8 ± 0.1 0.6–0.9
Nbss	68.2 ± 0.6 67.1–69.4	11.8 ± 1.7 9.4–13.9	4.4 ± 0.9 3.6–5.4	8.5 ± 0.5 7.7–9.2	4.5 ± 0.4 3.8–5.3	1.7 ± 0.3 1.2–2.0	0.5 ± 0.1 0.3–0.7	0.4 ± 0.1 0.2–0.6
Ti–rich Nbss	61.1± 1.6 59.6–64.5	22.7 ± 1.8 21.6–24.8	4.2 ± 1.1 3.1–5.6	5.5 ± 0.7 4.4–6.9	1.8 ± 0.4 1.0–2.4	3.0 ± 0.2 2.7–3.5	0.6 ± 0.1 0.4–0.7	1.1 ± 0.2 0.9–1.4
Nb_5_Si_3_	48.4 ± 0.3 47.7–48.6	7.8 ± 0.2 7.4–8.0	36.3 ± 0.6 35.4–37.2	3.9 ± 0.2 3.6–4.2	0.3 ± 0.1 0.1–0.5	0.5 ± 0.1 0.4–0.7	2.3 ± 0.1 2.1–2.6	0.5 ± 0.1 0.4–0.6
Ti-rich Nb_5_Si_3_	46.3 ± 0.7 45.1–47.0	11.9 ± 0.9 10.9–12.9	34.5 ± 0.7 34.0–35.7	2.9 ± 0.2 2.5–3.1	0.1	0.6 ± 0.1 0.5–0.7	2.8 ± 0.2 2.6–3.0	0.9 ± 0.2 0.7–1.1
Nb_3_Si	55.9 ± 0.4 55.2–56.1	8.0 ± 0.1 7.8–8.1	26.2±0.5 25.7–26.9	6.5 ± 0.2 6.2–6.8	1.5 ± 0.1 1.3–1.6	0	1.4 ± 0.1 1.4–1.5	0.5 ± 0.1 0.4–0.6
Eutectic	58.8 ± 0.6 58.0–59.8	10.8 ± 0.7 9.9–11.9	18.2 ± 0.6 16.1–18.1	6.2 ± 0.3 6.1–6.8	2.8 ± 0.2 2.7–3.2	1.3 ± 0.1 1.2–1.6	1.2 ± 0.2 0.9–1.5	0.7 ± 0.1 0.4–0.8
Heat-treated								
Large area	55.0 ± 1.3 53.3–56.9	12.0 ± 0.4 11.5–12.8	22.4 ± 1.6 20.1–24.5	5.2 ± 0.3 4.6–5.5	1.6 ± 0.3 1.1–2.1	1.2 ± 0.2 1.0–1.5	1.8 ± 0.2 1.5–2.1	0.8 ± 0.1 0.7–0.9
Nb_ss_	69.3 ± 0.5 68.7–69.8	12.3 ± 0.2 12.1–12.6	3.8 ± 0.7 3.1–4.7	8.0 ± 0.3 7.5–8.3	4.0 ± 0.2 3.8–4.3	2.3 ± 0.1 2.2–2.4	0.3 ± 0.1 0.2–0.3	0
Nb_5_Si_3_	48.3 ± 0.4 47.9–48.8	7.9 ± 0.2 7.7–8.2	35.9 ± 0.4 35.4–36.4	4.1 ± 0.1 3.9–4.2	0.4 ± 0.1 0.2–0.4	0.6 ± 0.1 0.5–0.7	2.3 ± 0.2 2.0–2.4	0.5 ± 0.1 04–0.5
Ti-rich Nb_5_Si_3_	44.8 ± 0.5 44.0–45.2	12.5 ± 0.4 12.1–13.0	35.3 ± 0.6 34.3–35.8	2.8 ± 0.2 2.4–3.1	0	0.7 ± 0.1 0.5–0.8	2.9 ± 0.3 2.7–3.3	1.0 ± 0.2 0.8–1.2

**Table 2:** Density of the as-cast (AC) alloys and % area of Nb_ss_ in the as-cast and heat-treated (HT) alloys JZ1 and JZ2.

Alloy	Density (g/cm^3^)	% Area Nb_ss_^a^		
		Top	Bulk	Bottom
JZ1-AC ^a^	8.24 ± 0.02 8.2–8.26	42.3 ± 0.7 41.3–43	46.5 ± 0.8 45.4–47.6	46.5 ± 1.0 45.4–48.2
JZ1-HT	-	-	46.5 ± 1.5 44–48.2	-
JZ2-AC ^a^	8.31 ± 0.02 8.28–8.33	32.9 ± 1.1 32.1–34.1	34.5 ± 1.0 33.9–35.6	21.9 ± 1.1 20.7–22.7
JZ2-HT	-	-	23.5 ± 2.7 20.4–25.4	-

^a^ includes the Nb_ss_ and Ti-rich Nb_ss_.

**Table 3:** The analysis data (at.%) for the alloy JZ2.

Area Phase	Nb	Ti	Si	Ta	W	Sn	Ge	Hf
As-cast								
Top	50.7 ± 0.9 49.0–50.5	11.6 ± 0.2 11.4–12.0	21.6 ± 1.7 19.2–23.7	5.4 ± 0.5 4.8–6.2	1.9 ± 0.2 1.7–2.1	2.6 ± 0.2 2.3–2.9	5.3 ± 0.4 4.8–5.9	0.9 ± 0.1 0.8–1.0
Bulk	50.3 ± 0.2 50.2–50.7	11.7 ± 0.4 11.3–12.4	21.7 ± 1.2 20.0–23.0	5.3 ± 0.3 5.0–5.7	2.0 ± 0.2 1.8–2.2	2.5 ± 0.3 2.1–2.8	5.4 ± 0.7 5.0–6.6	1.1 ± 0.1 0.9–1.3
Bottom	50.8 ± 0.9 50.1–52.1	12.1 ± 0.5 11.4–12.7	21.1 ± 1.7 18.8–22.5	4.9 ± 0.4 4.5–5.6	2.0 ± 0.2 1.9–2.4	3.2 ± 0.3 2.9–3.6	4.9 ± 0.2 4.7–5.1	1.0 ± 0.2 0.8–1.3
Nb_ss_	63.7 ± 0.7 62.9–64.7	11.5 ± 1.0 10.6–12.9	3.4 ± 0.4 3.1–4.1	9.3 ± 0.5 8.7–9.8	6.5 ± 0.4 5.9–7.0	4.1 ± 0.4 3.9–4.8	1.0 ± 0.2 0.8–1.2	0.5 ± 0.1 0.3–0.6
Ti–rich Nb_ss_	61.4 ± 0.9 60.3–62.4	15.5 ± 1.1 14.7–17.4	4.0 ± 0.5 3.5–4.7	7.6 ± 0.5 7.0–8.1	4.4 ± 0.4 3.7–4.7	5.5 ± 0.4 5.1–6.1	1.0 ± 0.1 0.8–1.2	0.6 ± 0.1 0.5–0.7
Nb_5_Si_3_	46.7 ± 0.2 46.4–47.0	8.2 ± 0.3 8.1–8.6	32.5 ± 0.7 31.4–33.2	4.3 ± 0.1 4.2–4.4	0.7 ± 0.1 0.6–0.8	1.0 ± 0.1 0.9–1.1	6.0 ± 0.2 5.9–6.2	0.6 ± 0.2 0.4–0.7
Ti–rich Nb_5_Si_3_	44.0 ± 1.1 42.1–45.1	13.0 ± 1.2 12.2–15.2	29.2 ± 0.8 28.3–30.4	3.1 ± 0.4 2.3–3.4	0.2	1.9 ± 0.4 1.5–2.5	7.6 ± 0.6 7.1–8.7	1.0 ± 0.1 0.9–1.2
A15	56.5 ± 0.9 55.4–57.8	16.8 ± 1.3 15.0–18.5	6.6 ± 0.5 5.8–7.1	4.7 ± 0.5 4.0–5.2	2.2 ± 0.4 1.7–2.7	9.5 ± 0.4 8.9–10.0	3.1 ± 0.2 2.9–3.4	0.6 ± 0.2 0.3–0.9
Eutectic	53.2 ± 0.9 52.2–54.3	13.5 ± 0.6 12.9–14.3	15.6 ± 0.4 15.1–16.1	5.9 ± 0.1 5.9–5.9	3.0 ± 0.2 2.9–3.0	3.4 ± 0.2 3.2–3.6	4.3 ± 0.2 4.2–4.6	1.1 ± 0.3 0.8–1.6
Heat-treated								
Large area	51.0 ± 0.6 50.3–51.8	12.0 ± 0.3 11.8–12.5	21.1 ± 1.1 19.5–22.6	5.4 ± 0.3 4.9–5.8	2.1 ± 0.2 1.8–2.2	2.7 ± 0.1 2.7–2.8	4.9 ± 0.2 4.6–5.2	0.8 ± 0.1 0.7–0.8
Nb_ss_	65.6 ± 0.3 65.3–66.2	10.7 ± 0.4 10.4–11.3	3.5 ± 1.0 2.5–4.9	10.0 ± 0.4 9.5–10.4	7.5 ± 0.2 7.3–7.7	2.2 ± 0.2 1.9–2.4	0.5 ± 0.1 0.4–0.6	0
Nb_5_Si_3_	46.7 ± 0.4 46.2–47.1	8.4 ± 0.5 7.9–9.1	32.3 ± 0.7 31.2–32.8	4.6 ± 0.2 4.4–4.8	0.7 ± 0.3 0.2–0.9	0.8 ± 0.1 0.8–1.0	5.9 ± 0.2 5.6–6.1	0.6 ± 0.1 05–0.7
Ti–rich Nb_5_Si_3_	37.9 ± 4.6 34.3–43.3	19.4 ± 4.6 14.3–22.8	28.6 ± 2.4 26.5–31.8	2.6 ± 0.3 2.2–2.9	0	0.7 ± 0.4 0.2–1.2	9.3 ± 2.1 6.8–10.9	1.5 ± 0.6 0.7–2.2
A15	58.8 ± 0.4 58.3–59.4	14.5 ± 0.3 14.2–14.8	5.7 ± 0.6 5.2–6.6	5.3 ± 0.2 4.9–5.4	2.3 ± 0.1 2.2–2.5	10.4 ± 0.2 10.2–10.7	2.9 ± 0.1 2.7–3	0

**Table 4:** Weight gains and oxidation rate constants of the alloys JZ1 and JZ2 after isothermal oxidation at 800 and 1200 °C for 100 h.

Alloy	800 °C		1200 °C		
	Weight Gain (mg/cm^2^)	Rate Constant	Weight Gain (mg/cm^2^)	Rate Constant	
		k_l_ (g cm^−2^ s^−1^)		k_l_ (g cm^−2^ s^−1^)	k_p_ (g^2^ cm^−4^ s^−1^)
JZ1	33.6 (100 h)	1.14 × 10^−7^ (0–58 h) 7.5 × 10^−8^ (58−100 h)	91.3 (100 h)	2.16 × 10^−7^ (9–100 h)	1.4 × 10^−8^ (0–9 h)
JZ2	28.9 (100 h)	1 × 10^−7^ (0–53 h) 6.8 × 10^−8^ (53–100 h)	71.7 (100 h)	1.8 × 10^−7^ (9–100 h)	5.5 × 10^−9^ (0–9 h)

**Table 5:** Analysis data (at.%) of phases in the alloy JZ2 after isothermal oxidation at 1200 °C for 100 h.

Area	Phase	O	Nb	Ti	Si	Ta	W	Sn	Ge	Hf
Oxide scale	Nb-rich oxide	74.4 ± 0.7 73.3–75.0	16.6 ± 0.9 15.6–17.7	5.7 ± 1.9 3.6–7.5	0.7 ± 0.5 0.3–1.4	1.8 ± 0.4 1.3–2.3	0.4 ± 0.2 0.3–0.7	–	–	0.3 ± 0.2 0.2–0.6
	Ti–rich oxide	73.8 ± 0.8 72.8–74.6	4.0 ± 0.7 3.5–5.2	20.9 ± 0.9 19.7–22.2	0.3 ± 0.2 0.1–0.5	0.6 ± 0.1 0.6–0.7	–	–	–	0.4 ± 0.1 0.3–0.4
	HfO_2_	71.8 ± 1.7 69.7–73.2	1.6 ± 0.4 1.2–1.9	1.1 ± 0.1 1.0–1.1	0.5 ± 0.9 0–1.6	–	–	–	–	24.9 ± 0.3 24.6–25.1
Diffusion zone 1	Nb_5_(Si_1–x_,Ge_x_)_3_	-	54.3 ± 0.9 52.8–54.8	1.6 ± 0.6 1.1–2.7	15.3 ± 0.5 14.6–15.7	5.2 ± 0.3 4.7–5.4	1.1 ± 0.2 0.9–1.4	-	22.4 ± 1.0 21.6–24.1	-
	W–rich Nb_5_(Si_1–x_,Ge_x_)_3_	-	47.4 ± 1.2 45.7–48.7	1.2 ± 0.2 1.0–1.5	12.8 ± 1.0 12.2–14.6	7.4 ± 0.3 7.2–7.9	6.2 ± 0.5 5.7–6.9	0.5 ± 0.4 0.3–1.0	24.8 ± 1.5 22.7–26.6	-
	NbGe_2_	-	33.0 ± 0.9 32.0–34.1	1.0 ± 0.5 0.2–1.4	4.4 ± 1.2 2.8–5.8	2.7 ± 0.4 2.3–3.4	0.9 ± 0.3 0.6–1.5	0.2 ± 0.1 0.1–0.4	57.8 ± 1.0 56.5–5.89	-
Diffusion zone 2	Nb_5_Si_3_	-	48.3 ± 0.3 47.8–48.7	8.0 ± 0.2 7.6–8.2	31.4 ± 0.7 30.7–32.4	4.5 ± 0.3 4.1–4.9	0.7 ± 0.1 0.6–0.9	1.0 ± 0.1 0.9–1.1	5.5 ± 0.2 5.3–5.7	0.6 ± 0.2 0.5–0.9
	Ti–rich Nb_5_Si_3_	-	45.8 ± 1.2 44.1–46.6	15.0 ± 1.8 13.7–17.7	24.9 ± 0.6 24.2–25.6	3.4 ± 0.5 2.7–3.7	0.3 ± 0.1 0.2–0.4	3.2 ± 0.4 2.8–3.7	6.8 ± 0.5 6.3–7.4	0.9 ± 0.1 0.7–1.0
	A15	-	61.0 ± 0.8 59.9–61.9	14.2 ± 1.0 12.9–15.4	4.7 ± 0.9 3.7–5.7	4.6 ± 0.3 4.2–4.9	2.0 ± 0.4 1.4–2.5	10.3 ± 0.3 10.0–10.8	3.2 ± 0.1 3.1–3.3	-
	Nb_ss_^a^	-	69.7	5.5	4.6	10.2	7.1	2.0	0.8	0.2
Bulk	Nb_5_Si_3_	-	47.0 ± 1.0 45.9–48.3	8.2 ± 0.2 8.1–8.6	31.9 ± 0.8 30.9–32.7	4.6 ± 0.3 4.2–5.0	0.7 ± 0.10 0.6–0.8	1.0 ± 0.1 0.9–1.1	5.9 ± 0.5 5.3–6.4	0.7 ± 0.1 0.5–0.8
	Ti–rich Nb_5_Si_3_	-	42.9 ± 1.5 40.6–44.6	17.0 ± 2.2 13.8–19.4	26.2 ± 0.9 25.0–27.3	2.9 ± 0.6 2.4–3.9	–	2.4 ± 0.4 1.8–2.9	7.6 ± 0.7 6.5–8.6	1.1 ± 0.1 1.0–1.3
	A15	-	58.9 ± 0.9 57.2–59.3	16.6 ± 0.5 16.2–17.3	3.9 ± 1.0 3.1–5.5	5.0 ± 0.4 4.6–5.6	2.0 ± 0.2 1.8–2.2	10.8 ± 0.5 10.4–11.7	2.8 ± 0.2 2.4–2.9	-
	Nb_ss_	-	66.1 ± 1.0 64.9–67.5	10.3 ± 0.4 9.7–10.7	3.9 ± 1.4 2.1–5.4	10.1 ± 0.5 9.6–10.9	6.8 ± 0.5 6.2–7.3	1.9 ± 0.6 1.2–2.7	1.0 ± 0.3 0.6–1.3	-

^a^ Ti oxide particles were formed in the phase, only two analyses were possible.

**Table 6:** Solutes in Nb-X binary alloys predicted to segregate to the surface.

Theory *	Element				
A	Si	Sn	Ge	Hf	Ti
B	-	Sn	Ge	-	Ti
C	Si	Sn	Ge	-	-
D	Si	Sn	Ge	Hf	Ti

* see text.
